# Bioactive lipids and allelopathic potential of the invasive plant *Heracleum sosnowskyi*: insights into its fatty acid composition, antimicrobial and cytotoxic effects

**DOI:** 10.3389/fphar.2025.1582694

**Published:** 2025-05-01

**Authors:** Eva Borska, Jorens Kviesis, Anna Ramata-Stunda, Vizma Nikolajeva, Linda Ansone-Bertina, Martins Boroduskis, Maris Klavins

**Affiliations:** ^1^ Department of Environmental Science, University of Latvia, Riga, Latvia; ^2^ Institute of Microbiology and Biotechnology, University of Latvia, Riga, Latvia

**Keywords:** *Heracleum sosnowskyi*, invasive plants, fatty acids, lipid metabolism, bioactivity, extraction, antimicrobial activity, cytoxicity

## Abstract

Sosnowsky’s hogweed (*Heracleum sosnowskyi* Manden) is one of the most dangerous invasive plant, notorious with presence of toxic substances. At the same time *H. sosnowskyi* phytochemistry from perspective of applications in pharmacology, especially lipids have not been much studied. This study aims to analyse lipids of *H. sosnowskyi*, especially fatty acids, their composition, metabolism patterns and biological activity. Extraction possibilities of lipids from different parts of *H. sosnowskyi* have been studied and besides traditional solvents, so called green solvents can be used. In lipid extracts various positional and geometric isomers of fatty acids have been found and their concentrations and profiles differ amongst plant parts. Multifactor statistical analysis demonstrates the contribution of the metabolism of fatty acids in different parts of a plant. *H. sosnowskyi* lipid extracts demonstrate high antimicrobial activity and cytotoxic activity against cancer cell lines and so plant biomass after eradication can be used to obtain substances with high application potential in the bio-pharma industry.

## 1 Introduction

Invasive plants can be considered a significant threat to biodiversity, agricultural production, and forestry and can even be dangerous to animals and humans (Gioria et al., 2023). Thus, activities to control their spreading and eradicate existing populations are needed and accordingly European Union legislation for several plants are mandatory (Booy et al., 2020; Frommelt, 2020).

As an example of plants of highest concern Sosnowsky’s hogweed (*H. sosnowskyi* Manden) can be mentioned. This plant from South -West of Asia as highly productive (height 3–5 m, according to estimations one plant produces up to 20,000 seeds), with high biomass and was introduced in Europe to serve as forage for cattle (Grzędzicka, 2022). However, this plant easily gets wild and presently occupies large territories in Eastern and Northern Europe as well as in other regions of the world (Cuddington et al., 2022; Grzędzicka, 2022) outcompeting indigenous species. Additional threats posed by *H. sosnowskyi* are related to the presence of phototoxic substances (furanocoumarins) in aerial parts of this plant. Skin contact with furanocoumarins after solar exposure can cause dermatitis, swelling, pain (Olennikov and Chirikova, 2023). The presence of furanocoumarins and other allelochemicals can be considered one of the key factors affecting the high competitiveness of *H. sosnowskyi* concerning other plant species as well as the danger posed by this and other Heracleum genus species (Grzędzicka, 2022). However, the phytochemical composition of *H. sosnowskyi* is far from explored and a group of substances with possibly high biological activity are lipids, comprising substances with low polarity, but represented by substances such as triglycerides, sterols, fatty acids and others. An in-depth understanding of invasive plant phytochemistry and their lipid composition is of key importance to understanding why some plants are invasive and can outcompete other plants (Paramonova et al., 2024).

Accordingly, according to European Union (EU) legislation (EU Regulation, 2025 No 1143/2014) Sosnowsky’s hogweed should be eradicated, but the removal of this plant raises another problem–what to do with abundant plant biomass. The main criteria for utilisation of invasive plant biomass include the safety of plant biomass utilisation and, the need to prevent further spreading, but of importance is to find ways how the plant biomass utilisation could be economically beneficial–can cover at least plant removal costs (Ceriani et al., 2023; Pušić et al., 2024).

Sosnowsky’s hogweed biomass (stems, leaves, roots flowers, seeds) contain many biologically active substances which can be isolated from plants and can find use in biomedicine, the food industry, as herbicides, in general in bioeconomy, for example, lipids (Paramonova et al., 2024). Another reason of Sosnowsky’s hogweed lipid study is needed to develop invasive plant phytochemical composition study, to understand plant metabolism, roles and functions of different phytochemicals as factors affecting their invasiveness. Better understanding of invasive plant phytochemistry could be a key factor to developing their control methods (Barrett et al., 2021; Jabeen et al., 2023; Poljuha et al., 2024).

This study aims to analyse the lipid composition of Sosnowsky’s hogweed, especially fatty acids, their composition, metabolism patterns and biological activity, to offer plant biomass safe utilisation possibilities after eradication and support understanding of invasive plant phytochemistry and their control possibilities.

## 2 Materials and methods

### 2.1 Materials

Reference standard substances of heptadecanoic acid, p-coumaric acid, palmityl alcohol, methyl heptadecanoate, citral, coumarin, tetracosane, oleanolic acid were purchased from Sigma-Aldrich Chemie Ltd (Steinheim, Germany) of analytical grade. Phenolphthalein (PhPh), N,O-bis(trimethylsilyl)trifluoroacetamide with trimethylchlorosilane (BSTFA/1% TMCS), 0.5 M trimethylphenylammonium hydroxide solution (TMPAH) in methanol for GC derivatization were purchased from the same producer. The dichloromethane (puriss p. a., ≥99.8%, Chem-Lab, Belgium), chloroform (puriss p.a., ≥99.9%, Lab-Scan, Poland), hexane (puriss p.a., ≥98%, Honeywell, Germany), methyl tert-butyl ether (MTBE puriss p. a., ≥99.9%, Sigma-Aldrich, Germany), ethyl acetate (EtOAc puriss p.a., ≥99.5%, Sigma-Aldrich, France) and dimethyl carbonate (DMC puriss p.a., ≥99%, Sigma-Aldrich, Germany) were of HPLC, GC grade. The purity of dimethyl sulfoxide (DMSO) was consistent with molecular biology grade.

### 2.2 Plant material and general extraction procedure

*H. sosnowskyi* plant and their parts (leaves, stems, flowers, seeds, roots) were sampled in the summer/autumn of 2023, 2024 in Asares parish, Jēkabpils district (Latvia) 56°08′04.4″N 25°55′34.2″E. *H. sosnowskyi* have been identified by the Botanic Garden of University of Latvia botanist PhD Laura Klavina and voucher specimens have been deposited at the Herbarium of the Department of Environmental Science (Herbarium number 2023. KLZ-1321) University of Latvia (Latvia). Wet plant biomass was stored at 10°C until processing. The biomass was dried for 24 h in an oven at 60°C. For analytical characterization before analysis, plant biomass was milled using a laboratory cutting mill (Fritsch Pulverisette 15) to a particle size <1 mm. Dried and ground parts of the plant were weighed into Whatman extraction thimbles and extracted with dichloromethane (CH_2_Cl_2_) (180 mL) in a Soxhlet extractor for 6 h at 40°C. The extracts were further reduced using a rotary evaporator at temperature not exceeding 35°C. The dry extracted lipids were refluxed with 5% KOH in MeOH for 3 h at 65°C and after adding 6 M HCl until pH 2.0 was reached, unsaponifiable lipids were extracted with diethyl ether. The lipid mixtures were methylated and then analyzed by GC-MS.

### 2.3 Conditions for selecting an appropriate extraction method

Several extraction methods were used considering results of previous and other studies (Boldor et al., 2010; Purmalis et al., 2025). On average, 1–3 g of air-dried sample from each plant part was weighed into an extraction thimble and extracted using Soxhlet, maceration, ultrasonic extraction and accelerated solvent extraction (ASE) methods using dichloromethane (CH_2_Cl_2_) as the solvent. The extraction by Soxhlet extractor (Behr Labor-Technik GmbH) was performed for 6 h at the boiling temperature of solvent. The obtained extracts were evaporated under reduced pressure and weighed. Maceration was performed using an orbital shaker (BioSan PSU U-20i) at room temperature. Approximately 1.00 g of seeds was poured into 30 mL of solvent and stirred at 150 rpm for 24 h. The resulting solution was filtered and evaporated. Ultrasound-assisted extraction was performed using ultrasonic bath (US bath, Bandelin Sonorex digiplus, Germany). Seeds weighing 1 g were poured with 10 mL of solvent and sonicated for 30 min until the temperature reached 40°C. The resulting solution was filtered, and the residue was treated with another dichloromethane portion. Overall, extraction was repeated 3 times. The ASE extraction was performed using a laboratory scale device Dionex ASE (Thermo Scientific). About 2.00 g of seeds were loaded into the extraction cell, followed by processing consisting of four repeated extraction cycles with a fresh portion of the solvent at 90°C, for 5 min per each cycle (4 cycles). From each experiment, 15 ± 2 mL of the extract was obtained.

### 2.4 Extraction solvent impact study

To obtain the highest dry residue (DW) values, the contribution of solvents of different Hansen solubility (HSP) was used. The HSP plays a significant role in determining how well solvent can interact with polar materials (Hansen, 2007). HSP total parameter (δ^2^, MPa^1/2^) for used solvents such as hexane (14.9), ethyl acetate (18.2), chloroform (19.0), dichloromethane (19.8), dimethyl carbonate (20.2), and methyl tert-butyl ether (20.7) were used comparing solvent extraction efficiency by Soxhlet extraction. The extraction results were reported in mg g^−1^ DW.

### 2.5 Chromatographic analysis of *H. sosnowskyi* lipids and fatty acids

#### 2.5.1 Gas chromatography-mass spectrometry (GC-MS) analysis

Chromatographic analyses were performed using a GC (Perkin Elmer, Clarus 580) coupled to a quadrupole MS (Clarus SQ 8°C) ESI source. The carrier gas (helium. 99.999% purity) had a programmed flow rate of 1.0 mL min^−1^ with a 1:4 split ratio (10 mL/min). The source was operated in the positive (ESI+) mode with an electron energy voltage of 70 kV. The total ion current mode was set in the m/z range 25–480, but the ion multiplier current was set to 1700 V with a scan time of 0.2 s (625 scans sec^−1^). The system was controlled by the “TurboMass Ver6.0.0” software and data processing was performed using the NIST MS 2.2 Library (FairCom Corp. United States) software.

#### 2.5.2 Lipid pre-column derivatization for GC-MS analysis

After removing the solvent below 60°C using a rotary evaporator, the unsaponifiable lipid extract (less than 2 mg) was resuspended in dry pyridine (0.5 mL), to which 0.02 mL of the BSTFA/TMSC reagent was added. This mixture was then maintained at 75°C for 30 min. Finally, 0.01 mL of methanol (MeOH) was added to the silylated solution to remove excess reagent. Final volumes should be adjusted according to the initial mass of the sample and lipid content. Quantitative data for tentatively identified compounds were obtained using the external standard method (ESM) with heptadecanoic acid (for carboxylic acids), p-coumaric acid (for aromatic carboxylic acids), palmityl alcohol (for alcohols), citral (for terpenes), coumarin (for coumarins), tetracosane (for alkanes), and oleanolic acid (for triterpenes). Reference compounds were selected based on their molecular ion fragmentation pathways being as close as possible to the fragmentation of the analytes.

#### 2.5.3 GC-MS conditions for lipid analysis after derivatizations

A capillary Elite-5MS column (PerkinElmer, 30 m × 0.25 mm, sorbent layer – 0.25 μm) was used as the stationary phase. The initial temperature of the column thermostat was 75°C (held for 2 min), then increased to 150°C (20.0°C min^−1^), then to 250°C (4.0°C min^−1^) and finally raised to 270 (4.0°C min^−1^). Analysis time 41 min. The MS ion source and transfer line temperature were 230°C and 290°C, respectively. The sample injector was at 290°C.

#### 2.5.4 Transesterification of fatty acids to methyl esters (FAME) for GC-MS analysis

To determine the fatty acid content in the extracts, the samples were treated with a methylation reagent according to (ASTMD5974 – 96, 1996) method. The extracts of plant parts were dissolved in 5 mL of diethyl ether, from which 20–600 µL was transferred into a 1.5 mL chromatography vial, and 1,000 µL of diethyl ether solution (MeOH/Et_2_O (1:1 v/v)), 10 mL of phenolphthalein solution (1% PhPh/MeOH, *w*/*v*), and 50 µL of 0.5 M TMPAH were added. The prepared solution was analyzed under gas chromatography conditions. The amount of each fatty acid methyl ester (FAME) was calculated from an ESM using a standard solution of methyl heptadecanoic acid.

#### 2.5.5 Fatty acid methyl ester GC-MS analysis conditions

Compounds were separated using an Omegawax 250 capillary column with a polyethylene glycol stationary phase (Supelco, 30 m × 0.25 mm. 0.25 μm film thickness). The column temperature program began at 75°C (held for 2.0 min) and increased to 130°C (20°C/min). Then to 205°C (4°C/min) and finally to 230°C (2.9°C/min), with a hold time of 14.88 min (total analysis time 48 min). The MS ion source temperature and transfer line temperature were 230°C and 260°C, respectively. The sample injector temperature was adjusted to 230°C. The retention indices RI (from non-linear, temperature-programming measurements) was defined by the following Equation 1:

$$\text{RI}=100\;\times \;\left(\left(1\;–\;w\right)\;\times \;f_{1}\left(t_{x}\right)+w\;\times \;f_{2}\left(t_{x}\right)\right),w=\left(t_{x}\;–\;t_{n}\right)/\left(\left(t_{n}+1\right)\;–\;t_{n}\right)$$ (1)

where *f*_1_ (*t*_*x*_) is a polynomial regression that is applied to three reference n-alkanes n−1, n, and n + 1; the second polynomial regression, *f*_*2*_ (*t*_*x*_), is applied to three reference n-alkanes n, n + 1, and n + 2. The interval between n and n + 1 is included in both polynomial regressions, covering all retention times in that range (Mjøs, 2004).

### 2.6 Antimicrobial activity testing

Microbial strains were obtained from the Microbial Strain Collection of Latvia (MSCL), University of Latvia, MIRRI-ERIC Consortium. Antimicrobial activity was assessed by two-fold serial broth microdilution method (Qaiyumi, 2007) against Gram-negative bacterium *Pseudomonas aeruginosa* MSCL 331, Gram-positive bacterium *Staphylococcus aureus* MSCL 334, yeast *Candida albicans* MSCL 378 and filamentous fungi *Fusarium oxysporum* MSCL 259 and *Pyrenophora tritici-repentis* MSCL 1625. Samples were dissolved in DMSO.

Mueller-Hinton broth (Biolife, Italy) was used for cultivation of bacteria and Malt extract broth (Millipore, Germany) was used for the cultivation of yeast and filamentous fungi. The inoculum of microorganisms was prepared in sterile water with a density of 0.08–0.10 at A625 and diluted 100-fold in appropriate broth. The 96-well plates were then incubated at 37°C with bacteria and C. albicans for 24 h or at 20°C with filamentous fungi for 48 h. The MIC was determined as the lowest concentration of the studied material, which showed no visible growth. From the wells where no microbial growth was detected, 4 µL of the liquid was plated on appropriate solidified media to determine MBC/MFC. Results are expressed as the median of three replicates.

### 2.7 Cytotoxicity testing

The cytotoxicity of the extracts was evaluated in Balb/c 3T3, HepG2, and A549 cell lines (all obtained from ATCC) as well as in primary dermal fibroblasts. Evaluation was done according to OECD testing guideline No. 129 (OECD, 2010) with some modifications. Balb/c 3T3, HepG2, and A549 cells were seeded into 96-well plates at a density of 8 × 10^3^ cells per well, while dermal fibroblasts were seeded at a density of 5 × 10^3^ cells per well. Cells were cultured in 100 μL of DMEM medium (Sigma, UK) supplemented with 1% penicillin (100 U/mL)–streptomycin (100 μg mL^−1^) (both Sigma, United States). 10% calf serum (Sigma, United States) was added to the media for Balb/c 3T3 cells, and 10% fetal bovine serum (Sigma, United States) was used for all other cell lines. The plates were incubated overnight at 37°C with 5% CO_2_ to allow cell attachment and initial proliferation. The extracts were dissolved in dimethyl sulfoxide (DMSO) (Sigma, UK) at a concentration of 200 mg mL^−1^ and filtered through a 0.2 μm syringe filter. Cells were rinsed with phosphate-buffered saline (PBS), and 100 μL of 5% serum-containing medium supplemented with the extracts at concentrations ranging from 0.0039 to 0.5 mg mL^−1^ was added to the wells. Cells grown in a medium without extracts or with the DMSO solvent served as controls. After a 48-h incubation at 37°C with 5% CO_2_, the cells were rinsed with PBS, and 250 μL of Neutral Red dye solution (25 μg mL^−1^, Sigma, UK) in culture medium was added to each well. The plates were incubated for 3 h at 37°C with 5% CO_2_. Following incubation, the cells were rinsed with PBS, and 100 μL of desorb solution [50% ethanol, 1% glacial acetic acid, 49% water (v/v/v)] was added to each well. The Neutral Red dye was extracted at room temperature for 15 min on a shaker. Absorbance at 540 nm was measured using a Tecan M200 Infinite Pro microplate reader (Tecan, Switzerland). Cell viability was calculated using the formula provided below. Curve fitting and IC_50_ calculations were performed using GraphPad Prism 9 software.

Cell viability was calculated using the following formula Equation 2:

$$\text{viability }\left(\%\right)=\frac{{Abs}_{540}\left(treatment\right)-{Abs}_{540}\left(background\right)}{{Abs}_{540}\left(untreated\;control\right)-{Abs}_{540}\left(background\right)}\times 100\%$$ (2)

### 2.8 Data treatment and analysis

The quantification of compounds from *H. sosnowskyi* could be carried out according to the following Equations 3, 4:

$$C_{X}=A_{X}\times C_{X}\times f_{1}\left(C_{X}\right)/A_{S}$$ (3)

$$\text{RRC}=f_{1}\left(C_{X}\right)=\frac{1}{2}\sum_{i=1}^{2}f_{1}^{\left(i\right)}\left(C_{X}\right)=C_{S}/C_{X}^{\left(i\right)}\times A_{X}^{\left(i\right)}/A_{S}$$ (4)

Where A_X_ and C_X_ were the peak areas and concentration of the analyte in sample solution, respectively, while A_S_ and C_S_ were the peak areas and concentration of the standard reference, respectively. The relative response coefficient (RRC) is calculated as the ratio of the concentration and peak area between the standard and the other analyte. The value for each analyte is obtained using two concentrations (the highest and lowest content), both of which fall within the calibration curve ranges. The data obtained in the study were expressed as the mean ± standard deviation (SD) values of at least three independent experiments. All statistical analyses were performed using one-way analysis of variance with Tukey’s Significant Difference (HSD) test using the JMP Pro statistical program and Pearson’s r Heatmap using the JASP statistical analysis program. Results were considered statistically significant at a p-value of less than 0.05. The fatty acid concentrations were calculated using the external standard method. Individual fatty acid components were identified based on their retention indices and chromatograms (Santercole et al., 2012; Alves and Bessa, 2009) and were compared with reference spectra in spectral libraries (NIST MS Search, Version 2.2).

## 3 Results

### 3.1 Lipid extraction condition selection

**FIGURE 1:**
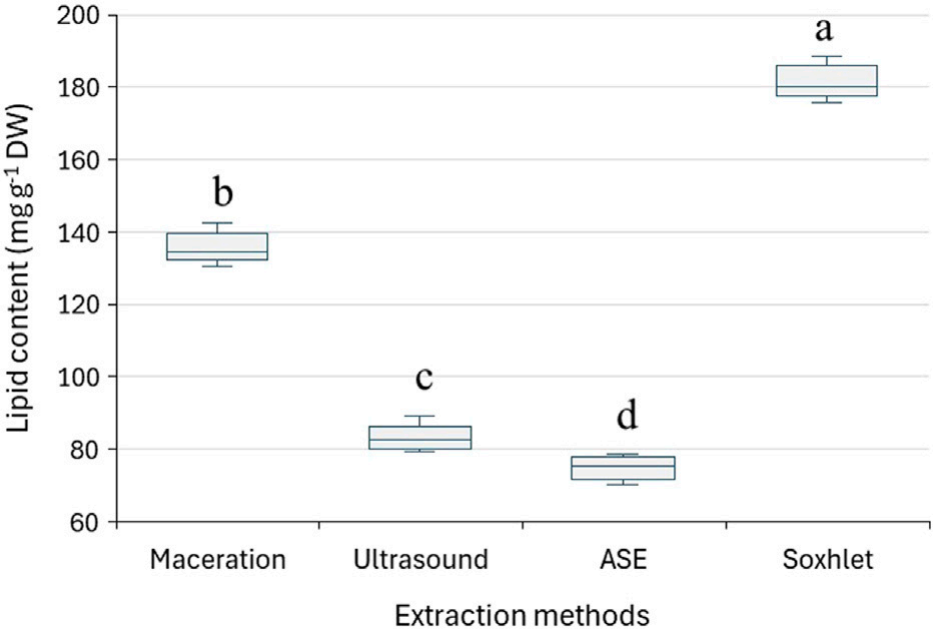
The effect of the extraction method on the yield of lipids in seeds of *H. sosnowskyi* (solvent – dichloromethane). The extraction time for quantitative recoveries by conventional method, ultrasonication, accelerated solvent extraction (ASE), and Soxhlet method were 1,440 min, 3 × 30 min, 4 × 5 min, and 360 min, respectively. The letters above each box represent the significance of the differences according to the Tukey HSD test at the 5% significance level. The parameters for created box diagram have been compiled in Supplementary Table S5.

Different extraction approaches were evaluated to determine the most effective method for lipid recovery. For this purpose, the seeds of Sosnovsky’s hogweed were used with CH_2_Cl_2_ as the solvent in all cases. Comparing the yield of extractives obtained using different extraction methods, it can be concluded that the Soxhlet extraction is the most effective (187 mg g^−1^), followed by the maceration method (136 mg g^−1^) but relatively less effective is the use of ultrasound-assisted extraction (83 mg g^−1^) (Figure 1).

Regardless of the chosen solvent, the yield of extractive substances in the seeds of *H. sosnowskyi* is significantly higher than the other analysed parts of the plant, that are presented in Table 1. The highest extractive yield (181.5 ± 7.1 mg g^−1^) from seeds was obtained using dichloromethane, while the lowest extractive yield was obtained using MTBE (97.7 ± 3.1 mg g^−1^) as a solvent. Solvents were evaluated according to the GlaxoSmithKline solvent sustainability guide (Alder et al., 2016).

**TABLE 1:** Extraction solvent impact on yield (mg g^−1^ mean ± SD) of lipids from different plant parts of Sosnovsky’s hogweed using Soxhlet extraction.

Parts of the plant	CH_2_Cl_2_	CHCl_3_	Hexane	MTBE	EtOAc	DMC
Seeds	181.5 ± 7.1	164.9 ± 6.4	114.9 ± 5.1	97.7 ± 3.1	127.1 ± 3.6	101.3 ± 6.2
Root	56.3 ± 3.3	48.8 ± 2.7	38.8 ± 1.4	63.5 ± 1.0	68.3 ± 2.1	58.2 ± 4.1
Stems	21.2 ± 4.0	19.3 ± 1.3	14.3 ± 1.7	18.7 ± 0.8	18.4 ± 0.9	43.7 ± 1.7
Blooms	71.9 ± 1.1	58.3 ± 4.2	56.8 ± 3.1	62.6 ± 1.6	53.4 ± 2.6	47.7 ± 2.4
Leaves	43.3 ± 3.4	26.3 ± 1.8	29.1 ± 2.2	29.5 ± 1.3	33.7 ± 1.1	24.0 ± 1.0

Based on the Hansen’s R_a_ criteria (Tirado et al., 2019; Peña-Gil et al., 2016), the solubility of potential lipids in the solvents used in the study was assessed. Considering that allelopathic properties were also found among representatives of fatty acids (Zhang et al., 2014; Bundit et al., 2021; Tsuzuki et al., 1987) and have not been sufficiently studied, representatives of this class were assessed for solubility using the Hansen test (see Table 2). The structure and physical-chemical properties of FAs gives that different solubility, which is often affected by the unusual position or absence of the double bond(s).

**TABLE 2:** The Hansen distance parameter R_a_ for fatty acids in various solutions which is calculated from reference values of dispersion (δd), polar (δp) and hydrogen-bonding (δh) contribution.

Fatty acid	R_a_ (MPa^1/2^)^a^					
	CH_2_Cl_2_	CHCl_3_	Hexane	MTBE	EtOAc	DMC
Stearic acid	4.29	3.03	7.44	3.29	2.46	6.58
Oleic acid	4.75	1.17	8.57	5.63	4.21	7.77
Linoleic acid	5.08	1.84	10.36	7.47	5.53	8.33
α-Linolenic acid	4.77	1.97	10.31	7.08	5.02	7.81

^a^ The output values of the Hansen parameters (HSP - δd, δp, δh) of the solvents were obtained from published sources (Bailón-Moreno et al., 2024; Rodríguez-Donis et al., 2018).

Considering that lower values characterize better solubility of substances (R_a_ < 10) in the solvent, judging by the R_a_ values in Table 2, the miscibility of fatty acids is ensured by chloroform, to a lesser extent by ethyl acetate and dichloromethane, and an average extent by MTBE, DMC and hexane. The observed results correspond to the lowest yield of extractables obtained using hexane as a solvent, and only slightly higher yields with DMC and MTBE.

### 3.2 Lipids in *H. sosnowskyi* plant parts

**FIGURE 2:**
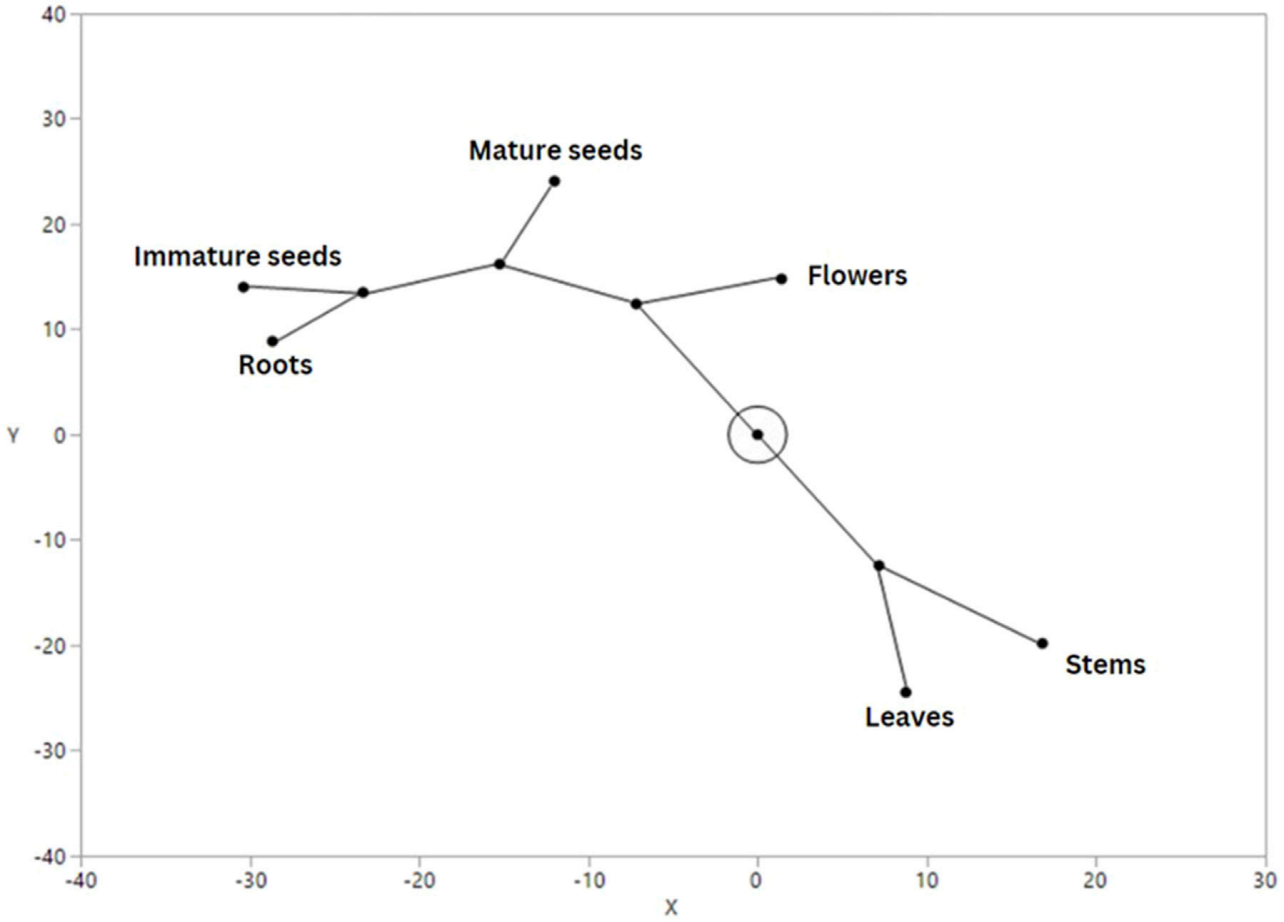
Extractive content of lipids belonging to different classes, obtained from different parts of hogweed (*H. sosnowskyi*) by means of extraction with dichloromethane. The x-axis represents the lipid content after log normalization, and the y-axis represents the lipid composition trait in different parts of the plant. Full lipid analysis results are provided in Supplementary Table S1 and extraction yields in Supplementary Tables S2, S3.

The stems of the hogweed have the lowest amount of total extractives, moreover, no aromatic carboxylic acids were detected, but small amounts of carboxylic acids [109.98 mg 100 g^−1^ DW; log (4.04 mg kg^−1^)], alcohols [0.24 mg 100 g^−1^ DW; log(1.38 mg kg^−1^)], and terpenes [1.66 mg 100 g^−1^ DW; log (2.22 mg kg^−1^)] were detected (Figure 2). It should be noted that the percentage of triterpenes in the stems (49%), compared to the total amount of extractives, is equivalent to flowers (53%) and leaves (56%). Among the reports studying *Heracleum mantegazzianum* species (Skalicka-Woź et al., 2017), the obtained hydrodistilled extract of fruits contained a high percentage of chromatographic peak area of carboxylic acid esters, which collectively showed lower efficacy in inhibiting *F. oxysporum* [MIC/(MBC/MIC) 7.1/(>30) mg mL^−1^] with ours (Table 3). This fact indicates some effect of esters on inhibiting fungal growth, which should be taken into account when formulating the extraction conditions and the desired activity effect. Although the MIC values of the stem extract are generally not as low as those of the flowers, they are comparable to those of the leaf extracts and in some cases show higher efficacy in testing against *C. albicans*, *F. oxysporum*, and the necrotrophic plant pathogen *P. tritici-repentis*. The effect of the triterpene content on the inhibition of microorganisms can be traced. Information can be found in the literature on the efficacy of the most common triterpenes in inhibiting several microorganisms (Catteau et al., 2018), which indicates a significant contribution to the maintenance of their antibacterial properties. According to a study (Bahadori et al., 2016) of the ethanol extract of fresh roots of Grey-Hairy hogweed (*Heracleum canescens*), sitosterol was detected in them (Bahadori et al., 2016). All parts of plants contained phytosterols, with β-sitosterol by far the major component (from 41% in roots to 69% in leaves of total sterols) among those present stigmasterol, campesterol, and schottenol. The last two phytosterols were absent in the roots, and campesterol was present only in the flowers among all the plant parts studied. The inhibitory effect of phytosterols such as stigmasterol and β-sitosterol on the growth *B. subtilis*, *E. coli*, and *C. albicans* has been reported (Alawode et al., 2021).

**TABLE 3:** Minimum inhibitory concentration (MIC)/and minimum bactericidal/fungicidal concentration (MBC/MFC) of Sosnowsky’s hogweed plant parts, mg mL^−1^, dissolved in DMSO. The DMSO concentration is expressed as a percentage. Results are expressed as the median of three replicates and shown in bold if the materials affect microorganisms (compared to the control).

Parts of the plants	Gram-positive bacteria	Gram-negative bacteria	Yeasts	Fungi	Fungi
	*S. aureus*	*P. aeruginosa*	*C. albicans*	*F. oxysporum*	*P. tritici-repentis*
Seeds	**0.24/1.95**	15.7/31.4	**0.98**/15.7	7.8/7.8	7.8/7.8
Roots	**0.15/2.3**	9.4/>18.7	**0.15/1.17**	4.7/4.7	4.7/4.7
Stems	**0.16/0.63**	**2.5/5.1**	**1.27**/2.5	**1.27**/2.5	**2.5/2.5**
Leaves	**0.06/0.49**	**2.0**/>7.8	**2.0**/3.9	**2.0**/3.9	3.9/3.9
Flowers	**0.14/0.14**	**9.0/17.9**	**0.56**/9.0	**1.12/4.5**	**2.2/2.2**
Control (DMSO)	12.5/>25	12.5/>25	12.5/12.5	12.5/12.5	12.5/12.5

The leaf extract contains high levels of triterpenes [741 mg 100 g^−1^ DW; log(4.87 mg kg^−1^)] and terpenes [58 mg 100 g^−1^ DW; log (3.76 mg kg^−1^)]. The previously established relationship suggests that the low MIC values obtained in microbiological tests are related to the presence of triterpenes in the extracts (Evaristo et al., 2014). The relatively low percentage of coumarins seems to limit the efficacy against more resistant microorganisms such as *P. aeruginosa* and *F. oxysporum*, although the root extract, which contains 45% coumarin, shows efficacy against the fungus *C. albicans*. Despite the fact that the content of alcohol compounds in leaf extracts is lower than in the flower extract, the leaves have the lowest MIC values against *S. aureus* (0.06 mg mL^−1^), indicating that alcohols are a significant, but not dominant, factor in their activity.

The flower extract contains a high concentration of triterpenes [1972 mg 100 g^−1^ DW (log 5.29 mg kg^−1^)]. The flowers showed high efficacy against most microorganisms, such as *S. aureus* (MIC 0.14 mg mL^−1^), *P. aeruginosa* (MIC 0.9 mg mL^−1^), and *C. albicans* (MIC 0.56 mg mL^−1^). Triterpenes are well known for their antibacterial and fungicidal properties (Catteau et al., 2018), which could be the main reason for the efficacy of the flowers.

The primary compounds found in the seed extracts were coumarins, furanocoumarins, hydrocarbons, alcohols, esters, and aldehydes. The predominant components of the oil were aliphatic esters (82.9%), followed by aliphatic alcohols (11%). The oil was mostly composed of octyl acetate (39.5%), hexyl 2-metylobutanoate (14.4%), hexyl 2-methylpropanoate (6%), hexyl butanoate (5.4%), and octanol (8.6%). Other components included octyl 2-methylobutanoate (4%), hexyl 3-methylobutanoate (2.6%), octyl 2-methylopropanoate (2.4%), hexanol (1.3%), hexyl acetate (1%), and octanal (0.7%) (Synowiec and Kalemba, 2015).

### 3.3 Antimicrobial activity and cytotoxicity of *H. sosnowskyi* lipids

All types of Sosnowsky’s hogweed samples (seeds, roots, stems, leaves and flowers) showed antimicrobial activity against several tested microorganisms (Table 3). All five samples inhibited the growth of *S. aureus* at concentrations ranging from 0.06 mg mL^-1^ (leaves) to 0.24 mg mL^−1^ for (seeds) and killed *S. aureus* at concentrations ranging from 0.14 mg mL^−1^ (flowers) to 2.3 mg/mL (roots). The Gram-negative *P. aeruginosa* was inhibited by seeds, stems, leaves and flowers, only at higher concentrations than *S. aureus*. All samples inhibited the growth of the eukaryotic microorganisms *C. albicans* and *F. oxysporum*, but only roots at 1.17 mg mL^−1^ were able to kill *C. albicans* and seeds, roots and flowers at 4.5–7.8 mg mL^−1^ were able to kill *F. oxysporum*. The least effect was on *P. tritici-repentis*. Seeds, roots and flowers had fungistatic and fungicidal effects at concentrations ranging from 2.2–7.8 mg mL^−1^.

The seed extract shows the highest amount of carboxylic acids [2,104 mg 100 g^−1^ DW (log 5.32 g kg^−1^)] and significant coumarin content [2,282 mg 100 g^−1^ DW; log(5.36 g kg^−1^)] but shows less effect against the microorganisms studied (MIC 0.24–15.7 mg mL^−1^). Comparison of data by MIC values to others (Walasek et al., 2015) report indicates the potential seeds extract to inhibit growth *C. albicans* (MIC/(MBC/MIC) 0.25/(2–4) mg mL^−1^) and *P. aeruginosa* (MIC/(MBC/MIC) 1.0/(>1) mg mL^−1^) in the context of furanocoumarins contents. High MIC values in seeds, among the studied plant parts, could indicate a different range of chemical compounds in the extracted substances by substance classes, regardless of their concentrations. In addition, differences in the chemical composition of seed extracts are noted in a study (Jakubska-Busse et al., 2013) between *H. sosnowskyi* and *H. mantegazzianum*, which indicates variations in carboxylic acids and their esters between varieties. The presence of aldehydes in the seed extract is associated with allelopathic activity (Mishynaa et al., 2015), but our extract did not contain representatives of the aldehyde and alcohol classes, which can be explained by a different extraction methodology. The percentage of triterpenes in the seed extract (13%), based on the total amount of extractives, was found to be four times lower than that in the flower extract (53%), which is directly reflected in the low antimicrobial activity.

The root extract is effective against *S. aureus* and *C. albicans* (MIC 0.15 mg mL^−1^), but less effective against other studied microorganisms (MIC 4.7–9.4 mg mL^−1^). The extract is distinguished by the highest furanocoumarin content (68%). For comparison, it should be noted that the percentage of furancoumarins in the hydrodistillation extract of root essential oils mentioned in the literature (Słowiński et al., 2024) reaches as much as 21.7%, compared to 22.9% compounds based on the p-isopropylmethylbenzene skeleton (p-cymene, thymol and carvacrol and their methyl esters etc.), which can be considered high in the context of essential oils. The antimicrobial activity of *Heracleum spp*. roots was previously attributed to furanocoumarins, which have inhibitory effects, especially on *Mycobacterium tuberculosis* (O'Neill et al., 2013). Although coumarins and carboxylic acids are abundant in the roots, as well as in the seeds and flowers, their biological activity against microorganisms appears to be lower, which is particularly reflected in the higher MIC values against *P. aeruginosa* (MIC 9.4 mg mL^−1^). The roots, with the second highest content of aromatic carboxylic acids (20%), have relatively good efficacy against the yeast species *C. albicans*, and have equivalent activity against pathogens. In general, root and seed extracts are less effective, although in the case of yeasts (e.g., *C. albicans*) they demonstrate significant inhibitory capacity.

Cytotoxicity was evaluated in the Balb/c 3T3 cell line, which is the standard cell line used for cytotoxicity assessments, as well as in two cancerous cell lines: the human hepatoma cell line HepG2 and the human alveolar carcinoma cell line A549. These results were compared to cytotoxicity observed in primary dermal fibroblasts. To compare the extracts, both IC_50_ values and non-cytotoxic concentrations (concentrations at which cell viability decreases by no more than 20%) were considered.

**FIGURE 3:**
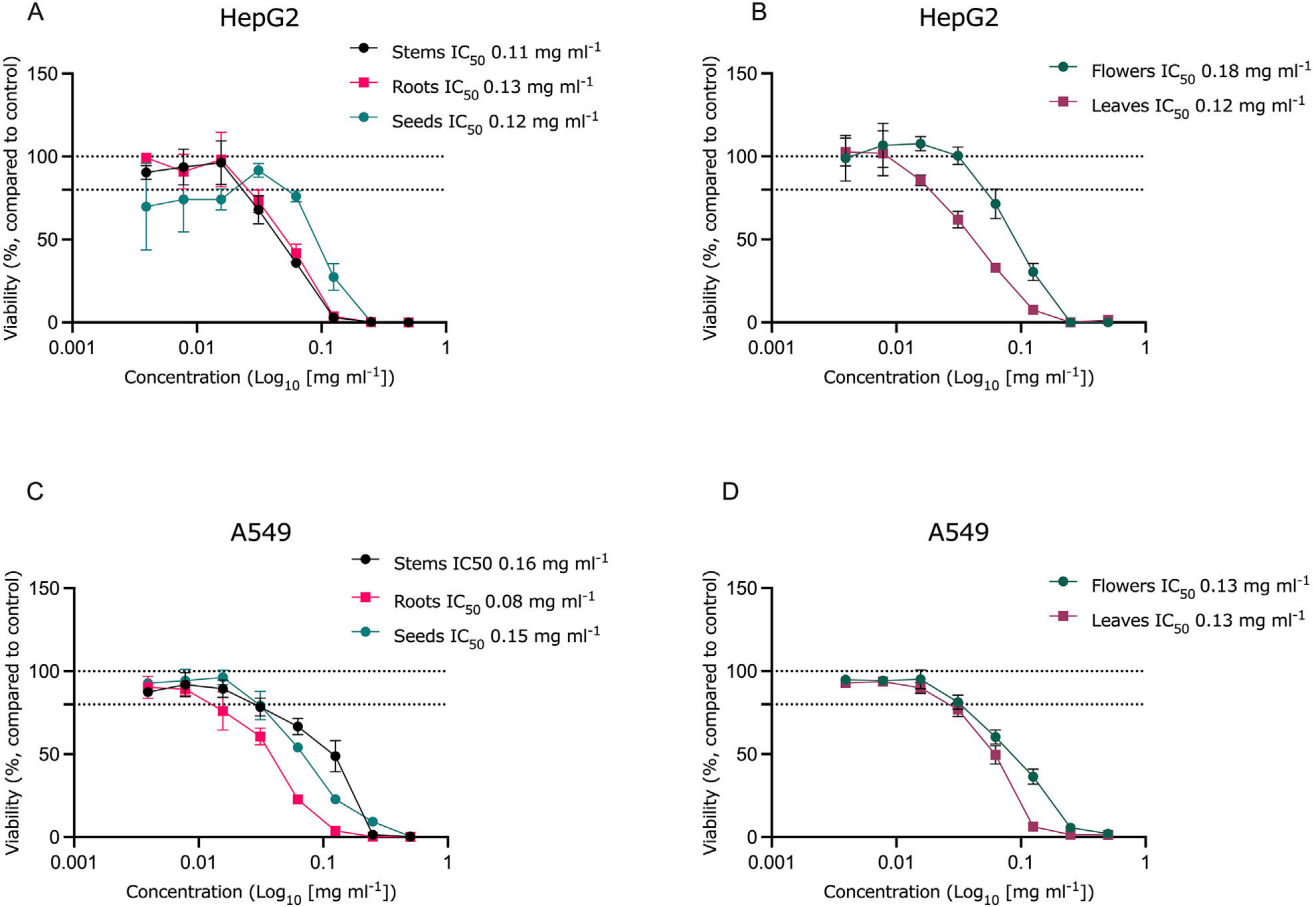
Cytotoxicity of Sosnovsky’s hogweed extracts in HepG2 **(A, B)** and A549 cell **(C, D)** cultures. Dotted lines represent the control level (100% viability) and 80% viability, n = 3.

**FIGURE 4:**
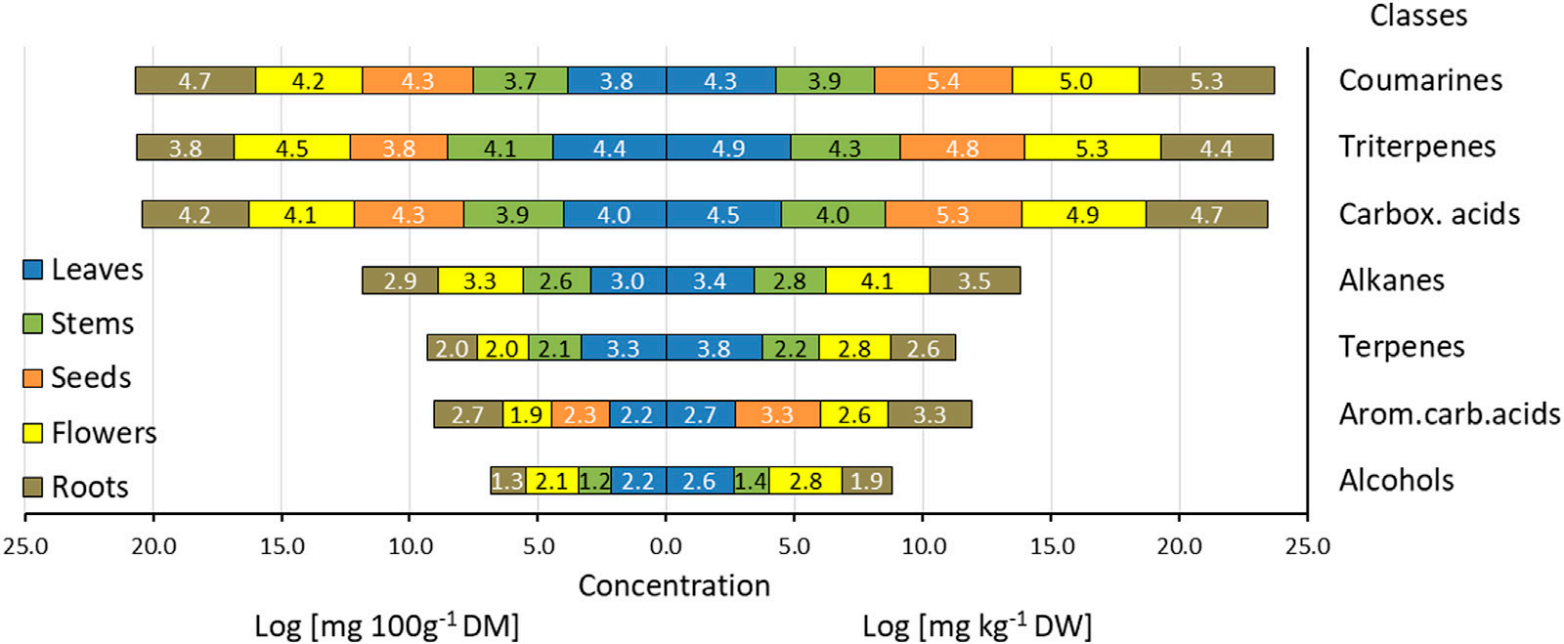
Constellation plot derived from hierarchical cluster analysis of fatty acid profiles in hogweed parts (*H. sosnowskyi*).

All extracts demonstrated similar levels of cytotoxicity across the tested cell lines (Table 4; Figures 3, 4). In the Balb/c 3T3 cell line, the extracts were slightly less cytotoxic compared to the other cell lines. Among the tested extracts, the root extract exhibited higher cytotoxicity than the other extracts in the Balb/c 3T3 and A549 cell lines. The leaf extract was the most cytotoxic in dermal fibroblasts, while the stem extract showed the highest toxicity in HepG2 cells.

**TABLE 4:** Cytotoxicity of Sosnovsky’s hogweed extracts on various cell lines. The highest non-cytotoxic concentration is the concentration (mg/mL) at which cell viability ^3^80%.

Parts of the plants	Balb/c 3T3		Dermal fibroblasts		HepG2		A549	
	IC_50_	Highest non-cytotoxic concen-tration	IC_50_	Highest non-cytotoxic concen-tration	IC_50_	Highest non-cytotoxic concen-tration	*IC*_*50*_	Highest non-cytotoxic concentration
Stems	0.14	0.031	0.16	0.031	0.11	0.016	0.16	0.016
Roots	0.11	0.031	0.16	0.031	0.13	0.016	0.08	0.0078
Seeds	0.18	0.031	0.20	0.063	0.12	0.031	0.15	0.031
Leaves	0.23	0.063	0.11	0.031	0.18	0.031	0.13	0.016
Flowers	0.21	0.063	0.18	0.016	0.12	0.031	0.13	0.016

### 3.4. *H. sosnowskyi* fatty acid profile

In the extracts obtained from various *H. sosnowskyi* biomass parts using CH_2_Cl_2_, a total of 38 fatty acids (FA) were identified (Table 5) and their chromatographic retention indices were determined. PUFA and MUFA together account for more than three-quarters of the total FA mass.

**TABLE 5:** Free fatty acid composition of the extract of the studied parts of *H. sosnowskyi* using GC-MS analysis.

Fatty acid	RI	Content of free fatty acids, µg 100g^-1^ DW					
		Roots	Stems	Leaves	Flowers	Immature seeds	Mature seeds
C8:0	1398	0.7 ± 0.0 ^b^	1.5 ± 0.0 ^a^	<0.1	0.7 ± 0.0 ^b^	0.3 ± 0.0 ^d^	0.4 ± 0.0 ^c^
Suc + C10:0	1609	0.3 ± 0.0 ^c^	3.3 ± 0.0 ^a^	1.6 ± 0.0 ^b^	0.3 ± 0.0 ^c^	<0.1	<0.1
C12:0	1817	0.4 ± 0.0 ^d^	1.7 ± 0.0 ^b^	4.1 ± 0.0 ^a^	1.5 ± 0.0 ^c^	<0.1	0.1 ± 0.0 ^e^
C14:0	2025	1.6 ± 0.0 ^d^	19.8 ± 0.1 ^b^	33.1 ± 0.3 ^a^	17.2 ± 0.1 ^c^	0.2 ± 0.0 ^f^	0.7 ± 0.0 ^e^
C9:0 9-oxo	2077	1.4 ± 0.0 ^bc^	23.2 ± 2.9 ^a^	3.3 ± 0.0 ^b^	0.7 ± 0.0 ^bc^	<0.1	<0.1
C15:0	2131	2.8 ± 0.1 ^c^	18.8 ± 0.1 ^a^	7.9 ± 0.3 ^b^	2.4 ± 0.0 ^d^	0.1 ± 0.0 ^f^	0.5 ± 0.0 ^e^
AzA	2154	1.6 ± 0.0 ^c^	10.2 ± 0.1 ^a^	4.8 ± 0.0 ^b^	1.2 ± 0.0 ^d^	<0.1	<0.1
C16:0	2236	60.1 ± 0.3 ^d^	884.7 ± 1.6 ^a^	615.8 ± 2.6 ^b^	137.3 ± 0.7 ^c^	3.4 ± 0.0 ^f^	42.1 ± 0.6 ^e^
C16:1n-11 (Δ^5c^)	2256	0.6 ± 0.0 ^c^	<0.1	1.8 ± 0.0 ^a^	0.5 ± 0.0 ^d^	0.1 ± 0.0 ^e^	1.2 ± 0.0 ^b^
C16:1n-9 (Δ^7c^)	2261	0.8 ± 0.0 ^de^	22.6 ± 0.9 ^a^	2.8 ± 0.0 ^c^	7.1 ± 0.0 ^b^	0.1 ± 0.0 ^e^	1.5 ± 0.0 ^d^
C16:1n-7 (Δ^9c^)	2270	0.5 ± 0.0 ^d^	2.6 ± 0.0 ^b^	7.0 ± 0.0 ^a^	0.6 ± 0.0 ^c^	0.1 ± 0.0 ^f^	0.3 ± 0.0 ^e^
C16:1n-5 (Δ^11c^)	2284	0.4 ± 0.0 ^b^	0.5 ± 0.0 ^a^	<0.1	0.3 ± 0.0 ^c^	<0.1	<0.1
C17:1n-7 (Δ^10c^)	2288	0.3 ± 0.0 ^b^	<0.1	<0.1	3.9 ± 0.1 ^a^	0.1 ± 0.1 ^c^	0.4 ± 0.0 ^b^
C16:1n-3 (Δ^12c^)	2288	0.3 ± 0.0 ^c^	6.8 ± 0.4 ^b^	86.0 ± 3.4 ^a^	1.1 ± 0.0 ^c^	<0.1	0.2 ± 0.0 ^c^
C16:2n-6 (Δ^7c,10c^)	2303	0.2 ± 0.1 ^d^	3.9 ± 0.1 ^b^	5.4 ± 0.0 ^a^	0.9 ± 0.0 ^c^	<0.1	<0.1
iC17:0	2306	<0.1	0.5 ± 0.0 ^a^	<0.1	<0.1	<0.1	<0.1
C17:0	2339	1.5 ± 0.0 ^d^	15.1 ± 0.1 ^b^	16.9 ± 0.2 ^a^	3.7 ± 0.0 ^c^	0.1 ± 0.0 ^f^	0.5 ± 0.0 ^e^
C16:3n-3 (Δ^7c,10c,13c^)	2370	0.9 ± 0.0 ^c^	55.0 ± 1.8 ^b^	633.2 ± 5.9 ^a^	6.3 ± 0.1 ^c^	<0.1	<0.1
C18:0	2450	5.3 ± 0.1 ^e^	45.4 ± 0.1 ^b^	84.8 ± 2.5 ^a^	18.8 ± 0.6 ^c^	1.1 ± 0.0 ^f^	11.3 ± 0.1 ^d^
C18:1n-9 (Δ^9t^)	2468	9.5 ± 0.1 ^e^	15.2 ± 0.2 ^d^	77.2 ± 1.9 ^a^	56.1 ± 0.1 ^b^	31.5 ± 0.0 ^c^	<0.1
C18:1n-7 (Δ^11t^)	2474	2.4 ± 0.0 ^d^	42.7 ± 0.1 ^b^	9.5 ± 0.9 ^c^	10.4 ± 0.1 ^c^	0.4 ± 0.0 ^d^	447.5 ± 5.4 ^a^
C18:1n-6 (Δ^12c^)	2479	7.6 ± 0.5 ^b^	6.5 ± 0.1 ^b^	15.9 ± 0.7 ^a^	1.6 ± 0.1 ^c^	<0.1	4.7 ± 0.4 ^b^
C18:1n-3 (Δ^14t^)	2490	1.2 ± 0.1 ^c^	2.6 ± 0.1 ^a^	2.2 ± 0.3 ^b^	0.5 ± 0.0 ^d^	0.6 ± 0.1 ^d^	1.4 ± 0.0 ^c^
C18:1n-2 (Δ^15t^)	2501	30.0 ± 1.9 ^a^	5.0 ± 0.6 ^a^	35.7 ± 0.5 ^a^	2.6 ± 0.1 ^a^	<0.1	<0.1
C18:2n-6 (Δ^9c,12t^)	2515	5.5 ± 0.4 ^b^	600.6 ± 13.2 ^a^	10.0 ± 0.8 ^b^	2.7 ± 0.0 ^b^	14.6 ± 0.1 ^b^	1.0 ± 0.0 ^b^
C18:2n-6 (Δ^9c,12c^)	2521	185.3 ± 1.9 ^c^	<0.1	856.1 ± 34.5 ^a^	230.5 ± 2.9 ^b^	<0.1	213.3 ± 2.3 ^bc^
C18:3n-3 (Δ^9c,12c,15c^)	2581	23.2 ± 0.8 ^d^	71.4 ± 1.1 ^c^	1687.4 ± 19.6 ^a^	135.5 ± 0.6 ^b^	0.3 ± 0.0 ^e^	3.6 ± 0.0 ^de^
(CLA) C18:2n-6 (Δ^10t,12c^)	2604	<0.1	<0.1	<0.1	<0.1	<0.1	4.6 ± 0.1 ^a^
C20:1n-[6-9] (Δ^14-11t^)	2652	0.5 ± 0.0 e	28.3 ± 0.2 ^a^	3.2 ± 0.0 ^c^	6.8 ± 0.3 ^b^	0.3 ± 0.0 ^e^	1.5 ± 0.0 ^d^
C20:1n-11 (Δ^9c^)	2668	<0.1	<0.1	<0.1	<0.1	0.1 ± 0.0 ^b^	1.5 ± 0.1 ^a^
C20:1n-9 (Δ^11c^)	2673	0.7 ± 0.0 ^d^	2.8 ± 0.0 ^c^	11.0 ± 1.0 ^a^	7.1 ± 0.2 ^b^	0.1 ± 0.0 ^d^	0.7 ± 0.0 ^d^
C20:1n-7 (Δ^13c^)	2683	<0.1	1.9 ± 0.1 ^b^	1.4 ± 0.1 ^b^	5.6 ± 0.2 ^a^	0.2 ± 0.0 ^d^	0.7 ± 0.0 ^c^
C22:0	2863	2.8 ± 0.0 ^c^	23.6 ± 1.9 ^a^	24.4 ± 0.3 ^a^	6.6 ± 0.0 ^b^	0.5 ± 0.0 ^d^	2.1 ± 0.1 ^cd^
C23:0	2965	1.2 ± 0.1 ^d^	17.0 ± 1.1 ^a^	13.4 ± 1.1 ^b^	3.3 ± 0.1 ^c^	<0.1	<0.1
C24:0	3070	5.2 ± 0.0 ^bc^	59.4 ± 4.1 ^a^	61.0 ± 5.8 ^a^	13.6 ± 1.2 ^b^	<0.1	<0.1
C25:0	3174	11.2 ± 0.0 ^a^	5.6 ± 0.3 ^a^	39.9 ± 5.1 ^a^	0.9 ± 0.1 ^a^	<0.1	0.2 ± 0.0 ^a^
C18:2n-6 (Δ^10t,12t^)(oxo-9)	3185	<0.1	4.2 ± 0.2 ^a^	<0.1	<0.1	<0.1	<0.1
C26:0	3260	2.2 ± 0.0 ^c^	20.7 ± 0.4 ^b^	41.5 ± 0.0 ^a^	2.6 ± 0.1 ^c^	0.4 ± 0.1 ^d^	<0.1
Σ MUFA		54.6 ± 2.9 ^cd^	113.8 ± 2.7 ^c^	255.8 ± 8.8 ^b^	98.2 ± 1.0 ^cd^	33.3 ± 0.2 ^d^	460.1 ± 6.0 ^a^
Σ PUFA		214.9 ± 3.1 ^d^	731.2 ± 16.2 ^b^	3186.7 ± 60.8 ^a^	375.0 ± 3.6 ^cd^	14.9 ± 0.1 ^e^	222.5 ± 2.4 ^d^
Σ SFA		98.9 ± 0.8 ^d^	1178.1 ± 13.0 ^a^	955.7 ± 18.2 ^b^	217.7 ± 3.2 ^cd^	6.4 ± 0.1 ^e^	59.6 ± 0.8 ^de^
UFA/SFA		2.7 ± 0.1 ^cd^	0.7 ± 0.0 ^e^	3.6 ± 0.1 ^c^	2.2 ± 0.0 ^d^	7.6 ± 0.2 ^b^	11.5 ± 0.2 ^a^
Unsaturation index		138.3 ^b^	84.3 ^d^	203.7 ^a^	143.4 ^b^	116.0 ^c^	122.4 ^c^

Data are expressed as the mean ± SD; RI, Retention indices for Omegawax250 capillary column (SD ± 3) which were calculated according to Equation 1; superscripts (a, b, c, d, e, f) - represent the significance of differences between values using the Tukey HSD, test (p < 0.05); MUFA, monounsaturated fatty acid; PUFA, polyunsaturated fatty acid; SFA, saturated fatty acids; AzA, azelaic acid; Suc, Succinic acid. Abbreviations used and full chemical names are provided in Supplementary Table S4. Fatty acid content expressed per mass of extract is included in Supplementary Table S6.

The results of the study showed a different range of fatty acids in the parts of the plant, of which high amounts linoleic acid (C18:2n-6; cis-isomer), α-linolenic acid (C18:3n-3) can be noted in the roots of hogweed, 9c, 12t-octadecadienic acid, lignoceric acid (C24:0) in the stems, roughanic acid (C16:3n-3, cis-isomer), α-linolenic acid (C18:3n-3), oleic acid (C18:1n-9) in the leaves and flowers, oleic acid, 9c, 12t-octadecadienic acid in immature seeds, and trans-vaccenic acid (C18:1n-7), α-linolenic acid prevail in mature seeds, while palmitic acid (C16:0) is present in comparable amounts in all parts of the hogweed. The major source of fatty acids found in leaves and flowers could mainly be considered monogalactosyldiacylglycerol, which usually forms one saturated (palmitic acid) and one unsaturated (palmitoleic acid) fatty acid acyl chain in the structure, or both unsaturated chains. Various positional and geometrical isomers of stearic acid (18:1n-9t; 18:1n-11t; 18:1n-14t; 18:1n-15t) are also found in parts of hogweed, including cis isomer (18:1n-12c), which has been found so far in Acer seed oils (Sun et al., 2018).

The leaves contained the highest level of FA at 4.398 mg per 100 g of dry-weight biomass. The stems and roots showed the greatest variety of fatty acids, featuring 33 different types. In contrast, mature seeds had the least diversity, with just 25 types, while immature seeds contained 23 types.

To investigate the biochemical differences in biomass samples collected during different developmental stages, the FA profiles of *H. sosnowskyi* seeds were analyzed. Immature seeds were gathered on 4 June 2023, and mature seeds were collected on 15 August 2023, from the same site. Comparative study revealed that both samples had twenty similar fatty acids. But whereas the mature seeds included five fatty acids absent from the other seed sample, the immature seeds contained three unique ones. These differences draw attention to important chemical variation in the biomass at several developmental phases.

Comparatively to immature seeds, mature seeds showed a significant increase in total FA content, underscoring their function as energy-dense storage tissues needed for germination and distribution. Reflecting the internal lipids rise throughout seed development, the concentration of C18:1n-7 (Δ11t), which in many vegetable oils is present in amounts up to 15%, drastically increase from 0.73% in immature seeds to 60.31% in mature seeds. Similarly, the polyunsaturated fatty acid C18:2n-6 (Δ9c12c), was absent in immature seeds but reached a concentration of 28.75% in mature seeds.

## 4 Discussion

Sosnowsky’s hogweed (*H. sosnowskyi* Manden) can be considered as one of the most notorious invasive plants (Mironova et al., 2022). *H. sosnowskyi* outcompete indigenous species and due to the presence of phototoxic substances (furanocoumarins) is dangerous to humans and animals (Egorov et al., 2021). However, the phytochemical composition of *H. sosnowskyi* is far from explored. An in-depth understanding of invasive plant phytochemistry and their lipid composition is of key importance to understand why some plants are invasive and can outcompete other plants. *H. sosnowskyi* should be eradicated, but the removal of this plant raises another problem – what to do with abundant plant biomass and wise plant biomass utilisation that could be economically beneficial – can cover plant eradication costs.

An important group of biologically active substances present in tissues of *H. sosnowskyi* are lipids and appropriate method to obtain them is extraction with solvents. Soxhlet extraction is preferable to maximize lipid yield, emphasizing its potential advantage over other extraction techniques. Comparing often-used traditional solvents, for example, CH_2_Cl_2_, with green solvents, for example, ethyl acetate and dimethyl carbonate, it can be concluded that for seeds of *H. sosnowskyi*, the extractive yield is slightly lower using environmentally friendly solvents while equivalent or slightly higher results are obtained by extracting other parts of the plant, such as roots and stems. Despite the contribution of green and conventional solvents to the extraction, the lowest yield of extractive substances was obtained for the plant stem. Regardless of the chosen solvent, the yield of extractive substances in the seeds of *H. sosnowskyi* is significantly higher than the other analysed parts of the plant. The obtained data show that, depending on the total yield of extractives, the solvents can be arranged in the following order CH_2_Cl_2_ > CHCl_3_ > EtOAc > DMC > MTBE > hexane.

Fatty acids are a significant element of plant metabolism. To understand their differences and functions in various plant parts chemometrical analysis has been used as it reveals plant metabolism character (Brown et al., 2009). Each tissue of *H. sosnowskyi* demonstrates a unique lipid and fatty acid profile closely aligned with its specific functional role. Reproductive tissues, such as seeds and flowers, are characterized by their richness in unsaturated fatty acids, which are essential for energy storage and critical for reproductive processes. In contrast, vegetative tissues, such as leaves and stems, are enriched in lipids that support their structural and photosynthetic functions. In total, 76 compounds (alkanes, alcohols, acids, coumarins, and phytosterols) were found in five studied *H. sosnowskyi* parts (Supplementary Table S1).

The correlation of fatty acid (FA) composition across various parts of *H. sosnowskyi* biomass is depicted in the Constellation Plot (Figure 4). The X and Y-axes show the first two principal components obtained from Principal Component Analysis (PCA), which together account for 78.6% of the variance in the dataset (Component 1: 50.1%; Component 2: 28.5%).

Compared to other biomass components (He and Ding, 2020; Rizzo et al., 2023; Petersen et al., 2024) stems and leaves have significant levels of SFA and PUFA, which is why they are positioned differently in the analysis. A key fatty acid involved in this process is palmitic acid (C16:0), which is crucial for maintaining the structural integrity of plant membranes and improving their fluidity and functionality (Lin et al., 2017). In the context of leaves, α-linolenic acid (C18:3n-3; Δ9c,12c, 15c) also plays an important role in lipid metabolism and functions not only as a potent antioxidant but also as a key component in the synthesis process (Zi et al., 2022). Remarkably, almost 70% of the fatty acids present in the leaves were categorized as PUFA, emphasizing their great potential for bioeconomic applications (Mititelu et al., 2025).

Most likely, 7c,10c,13c-hexadecatrienoic acid (C16:3n-3) found in the leaves and flowers of hogweed is one of the components of such pairs, which has so far been identified and isolated only from potato leaves after alkaline hydrolysis for the implementation of a study on recording plant responses to wounding (Weber et al., 1997). Among the plant parts studied, only the stem extract contained 9-oxooctadeca-10,12-dienoic acid (0.21%), which can significantly inhibit the fatty acid biosynthesis pathway of the Acetyl-CoA Carboxylase (ACC) enzyme, as indicated by the study of Watanebe et al. (1999). The presence of this fatty acid indicates that the plant may utilize it to control the activity of its ACC enzyme or release it into the environment to inhibit the growth of nearby plants, as it was found to exist as a free fatty acid. The results indicate that the stem of hogweed may be a significant source for herbicide production.

The plot reveals that mature seeds and flowers are clustered together, indicating a notable similarity in their fatty acid profiles. This resemblance could stem from shared biochemical functions, like energy-rich lipid storage or their involvement in seed dispersal. In contrast, immature seeds and roots are positioned towards the centre of the Constellation Plot, which suggests a transitional fatty acid profile. Immature seeds, which are still in the process of developing their lipid reserves, contain fewer unsaturated fatty acids but a higher proportion of saturated FA. Additionally, during seed maturation, a significant increase in the levels of C18:2n-6 (Δ9c, 12c) from <0.1 in immature seeds to 213.3 µg 100 g^-1^ DW in mature seeds and C18:1n-7 (Δ11t) from 0.4 in immature seeds to 447.5 µg 100 g^−1^ DW in mature seeds is observed.

**FIGURE 5:**
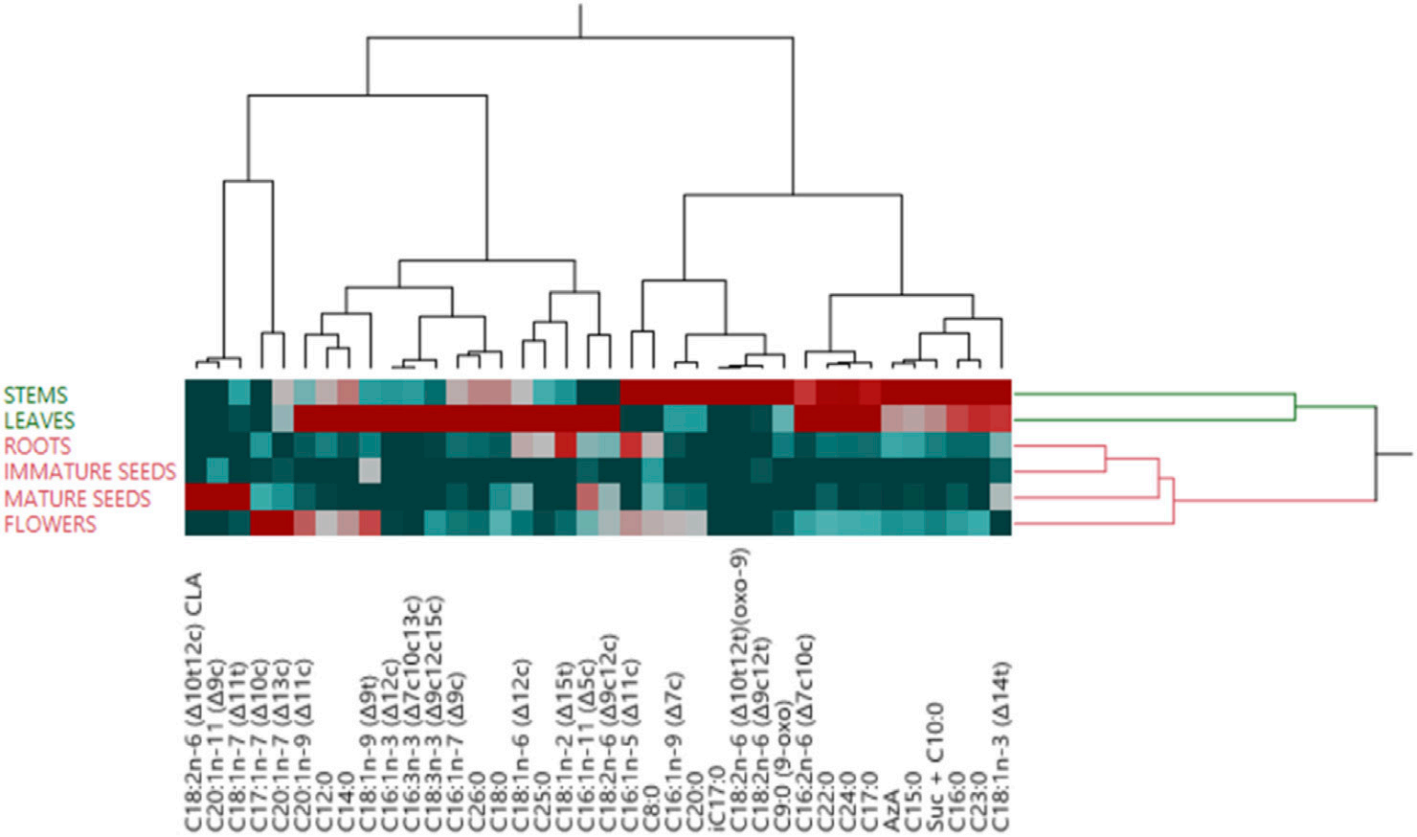
Pearson’s r heatmap of fatty acid (FA) profiles similarity based on extracts from six parts of hogweed (*H. sosnowskyi*).

Significant changes in the fatty acid (FA) profile of seeds are also confirmed by the Pearson’s r heatmap results (Figure 5), which display the Pearson correlation coefficients (indicating both the strength and direction of relationships) between different parts of the *H. sosnowskyi* plant. The findings indicate a strong positive correlation with significant statistical relevance between flowers and roots (0.886), leaves and flowers (0.784), and roots and leaves (0.523). This suggests that different parts of plants contain similar FA compounds and/or concentrations. Additionally, since there are no significant negative correlations, the analysis shows that the distribution of fatty acids across various plant components did not reveal strong inverse relationships. The most diverse FA profiles were observed between immature seeds and leaves, with a negative correlation coefficient of (−0.45).

**FIGURE 6:**
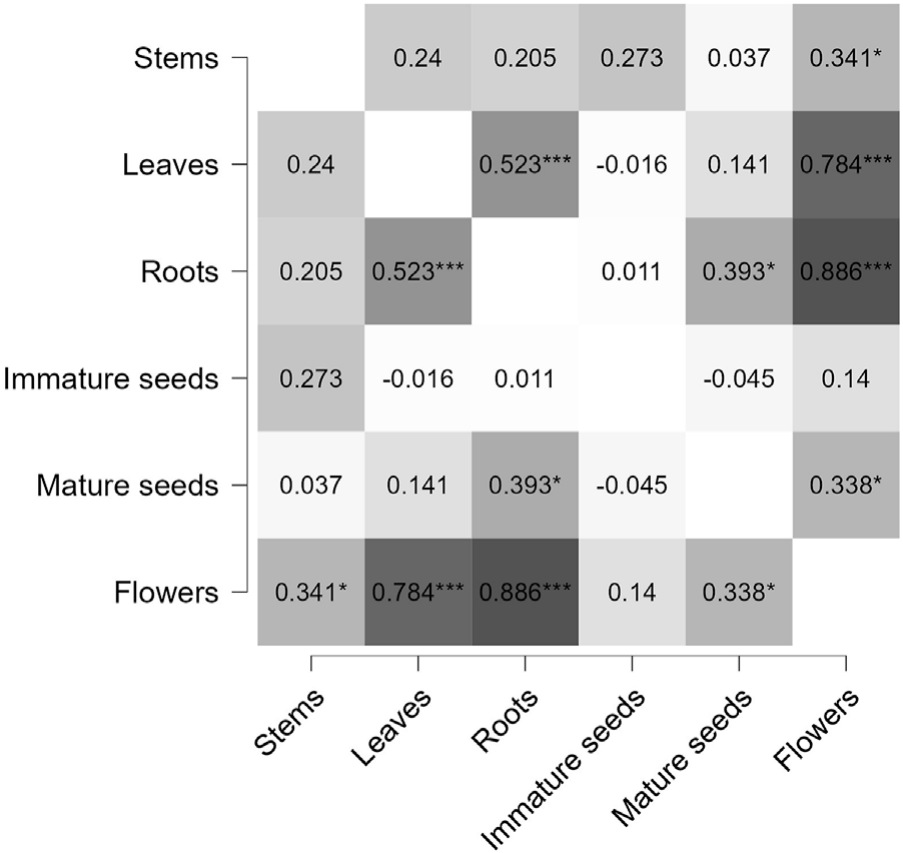
Dendrogram of hierarchical clustering analysis whit heatmap of fatty acid (FA) profiles similarity based on extracts from six parts of hogweed (*H. sosnowskyi*).

Dendrogram of hierarchical clustering analysis whit heatmap illustrates the different categories of tissues based on their fatty acid (FA) compositions (Figure 6). Similar to the Constellation Plot, the many biomass components of *H. sosnowskyi* can be broadly categorised into two primary clusters: one consisting of stems, leaves, and roots, and the other including seeds and flowers. Significantly, within the second cluster, the data indicate that flowers function as a connecting component, correlating with the fatty acid composition of the other plant parts. In this case, is able to see which FA and FA clusters most likely facilitate the clustering of biomass components and similar compositions. This underscores the common metabolic pathways and biochemical connections vital for reproduction. These studies show how functional difference among tissues is driven by fatty acid profiles, providing valuable insights for further research into lipid extraction for industrial, agricultural and pharmaceutical purposes.

The quantity of fatty acids discovered in this study much exceeds that previously documented in the literature about the chemical composition of *H. sosnowskyi* extracts. (Ivanova et al., 2015). These findings suggest that a higher total FA content in the extract or greater diversity of fatty acids does not necessarily correlate with an increase in the volume of extracted lipids.

Since the requirements of mature seeds for photosynthesis and transportation of substances are less acute than for immature seeds, significant changes in the mutual distribution of octadecenoic acid isomer pairs are observed. The results obtained show that the ss one of the fatty acids that can function as an indicator for evaluating the Initial assessment, seed sprouting rate, and viability C18:1n-9 content (Kaymak et al., 2022) in immature seeds decreases from 57.7% to an undetectable amount in mature seeds, while the C18:1n-7 content in them increases to 60.3% from the initially detected 0.73% in immature seeds, based on the total fatty acid content. For example, in canola oil, the C18:1n-7 has been, noted as a minor constituent, but it is a major constituent in the triglyceride of canola hulls (Appelqvist, 1969). It should be remarked that during the crushing of mature hogweed seed, the rudimentary endosperm may have mixed accompanied the seeds coat. Most likely, in the extract, the different morphological parts really represent tissue common to the seed.

Results illustrate the dynamic biochemical transformations that occur during seed maturation in Sosnowsky’s hogweed. These results provide important new perspectives on the metabolic adaptations that underlie seed maturation and their possible consequences in bioresource research.

*H. sosnowskyi* parts extracts differed in their inhibitory activity against yeast, 2 bacteria and 2 fungi. This integrated understanding of lipid and fatty acid profiles of *H. sosnowskyi* offers plant biomass safe utilisation possibilities after eradication and supports an understanding of invasive plant phytochemistry and their control possibilities.

The results show that all plant parts (seeds, roots, stems, leaves and flowers) have significant and broad antimicrobial activity against prokaryotic and eukaryotic microorganisms, especially against Gram-positive bacteria (*S. aureus*). Phytochemicals exert anti-staphylococcal activities mainly by destroying the membrane structure and inhibiting the efflux pump (Liang et al., 2022). Gram-negative bacteria, as in our case, are more resistant to inhibition by various plant extracts (Prasad et al., 2019). Crude extracts and/or compounds derived from plants are effective fungicides against a wide range of fungal species that cause illnesses in plants (Deresa and Diriba, 2023). Experiments have shown that extracts of *H. sosnowskyi* inhibit the growth of the soil-borne pathogen *F. oxysporum*, which causes plant root rot and wilt (Cai et al., 2022) and *P. tritici-repentis*, which causes tan spot of wheat (Andersen et al., 2021).

**FIGURE 7:**
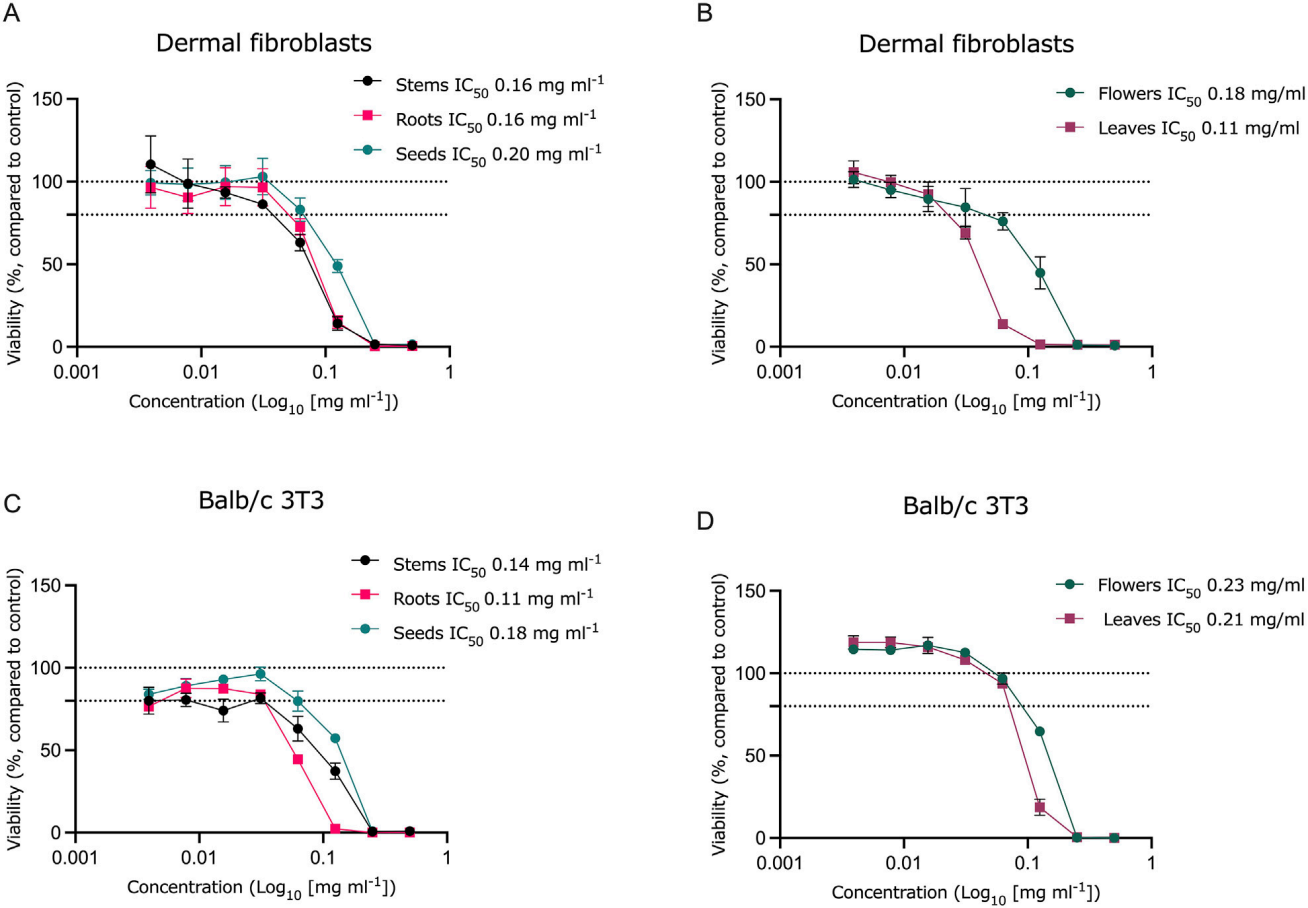
Cytotoxicity of Sosnovsky’s hogweed extracts in dermal fibroblast **(A, B)** and Balb/c 3T3 cell **(C, D)** cultures). Dotted lines represent the control level (100% viability) and 80% viability, n = 6 for dermal fibroblasts, n = 3 for Balb/c 3T3.

Although all extracts exhibited comparable cytotoxicity across the tested cell lines, slight variations in the responses were observed between the cell lines. Lower concentrations of the stem, root, seed, and flower extracts were required to induce cytotoxic effects in the cancerous cell lines HepG2 and A549. However, the observed differences and cell-type specificity of the extracts cannot be conclusively determined from these results. At the same time, high cytotoxic activity against cancer cell lines A549 and HepG2 is promising to further explore the anticarcinogenic activity of compounds extracted from *H. sosnowskyi* (Figures 3, 7).

Cytotoxicity results must be carefully considered when evaluating the potential antimicrobial applications of the extracts. From our findings, it can be observed that only the minimal inhibitory concentration of the leaf extract falls within the non-cytotoxic concentration range.

The effects of plant extracts combine the effects of various compounds. Since the study shows that different plant parts differ in the composition of biologically active substances, differences in composition can explain the different effects of plant parts on microorganisms.

## 5 Conclusion

The combined studies of the constellation plot, dendrogram, and heatmap provide a complete knowledge of the chemical variation inherent in *H. sosnowskyi* tissues. Every tissue shows a different fatty acid profile closely matched to its specific functional role. Rich in unsaturated fatty acids - which are vital for energy storage and fundamental for reproductive processes - reproductive tissues including seeds and flowers define themselves. By contrast, vegetative tissues - such as stems and leaves are enhanced in lipids that support their structural and photosynthetic roles.

In total, 76 compounds (alkanes, alcohols, acids, coumarins, and phytosterols) were found in five hogweed parts in addition the hogweed parts extracts differed in their inhibitory activity against yeast, two bacteria and two fungi. All extracts exhibited comparable cytotoxicity across in the Balb/c 3T3, HepG2 and A549 cell lines, only the minimal inhibitory concentration of the leaf extract falls within the non-cytotoxic concentration range. Of the plant parts only the stems contain 9-oxooctadeca-10,12-dienoic acid in free forms, which may acts as an inhibitor of the reaction in which acetyl-CoA is converted to malonyl-CoA and affects fatty acid biosynthesis.

These results provide important new perspectives on the biochemical functions of *H. sosnowskyi* plant components, forming the basis for practical applications as well as demonstrating the pharmacological potential of plant extracts. With their high PUFA content, leaves offer the possibility for bioactive chemical extraction, which might be used in nutraceutical or pharmaceutical industries. On the other hand, stems provide chances for use in industrial processes such the synthesis of structural biomaterials or biofuels since of their high concentrations of saturated fatty acids. This combined knowledge of fatty acid profiles emphasizes the importance of *H. sosnowskyi* as a source of several research and industrial applications.

## Data Availability

The datasets presented in this study can be found in online repositories. The names of the repository/repositories and accession number(s) can be found in the article/Supplementary Material.
