# Supplementary material for: Bioactive lipids and allelopathic potential of the invasive plant Heracleum sosnowskyi: insights into its fatty acid composition, antimicrobial and cytotoxic effects
Source: Front Pharmacol. 2025 May 1;16:1582694. doi: 10.3389/fphar.2025.1582694 (PMC12078124; doi:10.3389/fphar.2025.1582694)
Supplement: Supplementary file 1 [file DataSheet1.docx]

Table S1. Lipid extract composition and content (mg 100 g^-1^ DW) of *H. sosnowskyi* by GC-MS.

| **No.** | **Compound (TMS derivative)** | **RI** | **Leaves** | **Stems** | **Mature seeds** | **Flowers** | **Root** |
| --- | --- | --- | --- | --- | --- | --- | --- |
| 1 | Oleyl alcohol | 2162 | 0.207 ± 0.004 | – | – | – | – |
| 2 | Falcarindiol | 2323 | − | – | – | – | 0.851 ± 0.001 |
| 3 | 1-Hexacosanol | 2920 | 1.844 ± 0.003 | 0.237± 0.006 | – | 5.952 ± 0.004 | – |
| 4 | 1-Octacosanol | 3139 | 1.911 ± 0.037 | – | – | 1.104 ± 0.165 | – |
| 5 | 1-Triacontanol | 3350 | 0.370 ± 0.004 | – | – | – | – |
| 6 | n-Heptadecane | 1701 | – | 0.645 ± 0.002 | – | – | – |
| 7 | Pentacos-1-ene | 2489 | – | – | – | 2.881 ± 0.052 | – |
| 8 | n-Pentacosane | 2501 | 0.976 ± 0.024 | – | – | 11.51 ± 0.28 | – |
| 9 | n-Hexacosane | 2600 | – | – | – | 1.363 ± 0.010 | – |
| 10 | Heptacos-1-ene | 2689 | – | – | – | 3.917 ± 0.129 | – |
| 11 | n-Heptacosane | 2700 | 2.194 ± 0.013 | – | – | 22.51 ± 0.10 | – |
| 12 | n-Octacosane | 2800 | – | – | – | 2.848 ± 0.190 | – |
| 13 | n-Nonacosane | 2900 | 7.046 ± 0.033 | – | – | 23.206 ± 0.350 | – |
| 14 | n-Triacontane | 3000 | – | – | – | 2.268 ± 0.095 | – |
| 15 | n-Hentriacontane | 3100 | 12.11 ± 0.08 | – | – | 30.569 ± 0.761 | – |
| 16 | n-Dotriacontane | 3200 | – | – | – | 2.456 ± 0.191 | – |
| 17 | n-Tritriacontane | 3302 | 2.358 ± 0.021 | 5.441 ± 0.021 | – | 12.352 ± 0.206 | – |
| 18 | n-Tetratriacontane | 3404 | 1.341 ± 0.027 | – | – | – | – |
| 19 | (E)-2-Heptenoic acid | 1211 | 0.329 ± 0.011 | – | – | – | – |
| 20 | Benzoic Acid | 1249 | 2.568 ± 0.019 | – | 10.43 ± 0.34 | 1.488 ± 0.019 | 9.791 ± 0.143 |
| 21 | Octanoic acid | 1260 | 6.785 ± 1.579 | – | 29.49 ± 1.33 | 4.374 ± 0.095 | 11.68 ± 0.11 |
| 22 | Benzeneacetic acid | 1305 | – | – | – | 1.006 ± 0.005 | – |
| 23 | p-Toluic acid | 1359 | – | – | – | – | 2.006 ± 0.042 |
| 24 | Nonanoic acid | 1365 | 1.159 ± 0.760 | – | 6.017 ± 0.374 | 1.603 ± 0.027 | 3.179 ± 0.005 |
| 25 | Decanoic acid | 1464 | 6.030 ± 0.454 | – | 3.378 ± 0.025 | 3.146 ± 0.168 | 4.570 ± 0.267 |
| 26 | p-Anisic acid | 1534 | 0.025 ± 0.036 | – | – | – | 6.657 ± 7.837 |
| 27 | Undecanoic acid | 1562 | 1.197 ± 0.058 | – | – | – | 2.608 ± 0.113 |
| 28 | Dodecanoic acid | 1659 | 0.670 ± 0.178 | – | 1.700 ± 0.043 | 1.961 ± 0.392 | 2.793 ± 0.011 |
| 29 | Suberic acid | 1707 | 0.308 ± 0.123 | – | – | 0.252 ± 0.017 | – |
| 30 | 6-Phenylhexanoic acid | 1738 | 1.936 ± 0.057 | – | – | 1.625 ± 0.150 | 0.199 ± 0.028 |
| 31 | Tridecanoic acid | 1748 |  | – | 1.372 ± 0.195 | – | 1.285 ± 0.021 |
| 32 | Myristic acid | 1856 | 7.324 ± 0.057 | – | 8.939 ± 0.179 | 35.53 ± 0.02 | 18.077 ± 0.077 |
| 33 | Quininic acid | 1861 | 0.902 ± 0.008 | – | – | – | – |
| 34 | Phenyloctanoic acid | 1943 | 0.271 ± 0.454 | – | 11.34 ± 0.05 | 0.342 ± 0.027 | – |
| 35 | Pentadecanoic acid | 1956 | 1.712 ± 0.047 | – |  | 1.761 ± 0.015 | 20.74 ± 0.07 |
| 36 | Palmitelaidic acid | 2023 | 1.153 ± 0.336 | – |  | 3.970 ± 0.107 | 5.368 ± 0.250 |
| 37 | Palmitic Acid | 2054 | 204.2 ± 0.6 | – | 960.1 ± 2.5 | 402.5 ± 0.3 | 448.3 ± 0.3 |
| 38 | (Z)-10-Heptadecenoic acid | 2137 | 4.246 ± 0.0034 | – | 24.83 ± 0.83 | – | – |
| 39 | Margaric acid | 2150 | 0.470 ± 0.010 | – | 9.029 ± 0.111 | 3.149 ± 0.069 | 4.452 ± 0.070 |
| 40 | Linoleic acid | 2219 | 0.804 ± 0.001 | – | 9.686 ± 0.259 | 57.796 ± 1.501 | – |
| 41 | Oleic Acid | 2224 | 15.701 ± 0.200 | – | 838.2 ± 0.2 | 152.3 ± 1.0 | – |
| 42 | α-Linolenic acid | 2230 | 4.934 ± 0.054 | – | 12.376 ± 0.096 | – | – |
| 43 | Petroselinic acid | 2234 | 16.382 ± 0.063 | – | – | – | – |
| 44 | Stearic acid | 2254 | 7.340 ± 0.180 | – | 83.01± 0.13 | 234.1 ± 0.7 | 31.78 ± 0.90 |
| 45 | 9,12-Octadecadiynoic acid | 2268 | – | – | 2.493 ± 0.064 | 1.103 ± 0.057 | – |
| 46 | 7-Nonadecenoic acid | 2295 | 0.685 ± 0.003 | – | 10.32 ± 0.05 | 0.950 ± 0.045 | – |
| 47 | Unknown fatty acid 1 | 2309 | 11.04 ± 0.19 | – | – | 4.556 ± 0.140 | – |
| 48 | 10-Nonadecenoic acid | 2335 | – | – | 2.388 ± 0.008 | 0.396 ± 0.032 | – |
| 49 | 3-Hydroxy-octadecanoic acid | 2377 | 4.059 ± 0.024 | 1.145 ± 0.104 | – | 6.469 ± 0.037 | – |
| 50 | 11-Eicosenoic acid | 2422 | – | – | – | 0.744 ± 0.0.283 | – |
| 51 | Eicosanoic acid | 2449 | – | – | – | 2.807 ± 0.091 | – |
| 52 | Unknown fatty acid 3 | 2478 | – | – | 31.52 ± 0.95 | – | – |
| 53 | Unknown fatty acid 4 | 2538 | – | – | – | 2.619 ± 0.005 | – |
| 54 | Unknown fatty acid 5 | 2544 | – | – | – | 1.590 ± 0.103 | – |
| 55 | Unknown fatty acid 6 | 2555 | – | – | 43.16 ± 0.03 | – | – |
| 56 | Behenic acid | 2645 | – | – | 8.978 ± 1.244 | 2.689 ± 0.416 | – |
| 57 | 3-Hydroxydocosanoic acid | 2692 | – | – | – | 0.654 ± 0.080 | – |
| 58 | Ficusin | 1798 | 122.7 ± 0.2 | 4.748 ± 0.522 | 1309 ± 3 | 333.6 ± 0.7 | 220.9 ± 0.6 |
| 59 | Umbelliferone | 1849 | – | – | – | 7.693 ± 0.183 | – |
| 60 | Isopsoralen | 1856 | – | – | – | 18.19 ± 0.40 | – |
| 61 | Isobergapten | 2040 | – | – | – | – | 101.5 ± 2.2 |
| 62 | Methoxsalen | 2048 | 4.925 ± 0.692 | 17.67 ± 0.136 | 263.8 ± 2.4 | 252.9 ± 8.5 | 220.7 ± 2.4 |
| 63 | Bergapten | 2076 | 15.84 ± 0.84 | 6.958 ± 0.100 | 308.7 ± 1.2 | 95.19 ± 1.14 | 276.8 ± 0.5 |
| 64 | Falcarinol | 2104 | – | – | – | – | 24.74 ± 0.72 |
| 65 | Pimpinellin | 2140 | – | 1.420 ± 0.006 | 195.3 ± 3.8 | 21.03 ± 6.27 | 729.8 ± 0.3 |
| 66 | Isopimpinellin | 2241 | – | – | 205.0 ± 4.0 | 165.0 ± 6.8 | 288.0 ± 0.8 |
| 67 | Neophytadiene | 1845 | 10.40 ± 0.28 | – | – | – | – |
| 68 | Hexahydrofarnesyl acetone | 1851 | – | – | – | – | – |
| 69 | Phyta-1,3(E)-diene | 1871 | 2.312± 0.110 | – | – | – | – |
| 70 | 3,7,11,15-Tetramethyl-2-hexadecen-1-ol | 1889 | 2.478± 0.063 | – | – | – | – |
| 71 | Phytol | 2182 | 37.06± 0.98 | – | – | 1.665 ± 0.013 | 3.567 ± 0.137 |
| 72 | Unknown terpene 1 | 3361 | 5.959± 0.163 | – | – | 4.171 ± 0.364 | – |
| 73 | Campesterol | 3242 | – | – | – | 83.55 ± 5.32 | – |
| 74 | Stigmasterol | 3270 | 139.5± 0.1 | – | 145.7 ± 3.9 | 467.1 ± 6.7 | 148.4 ± 1.5 |
| 75 | β-Sitosterol | 3329 | 505.0± 4.8 | – | 380.9 ± 0.3 | 1258.325 ± 15.413 | 104.9 ± 1.8 |
| 76 | Schottenol | 3391 | 96.45± 4.60 | – | 157.1 ± 3.0 | 163.1 ± 4.6 | – |

Table S2. Lipid content (mg g^-1^ DM) in different parts of the hogweed (*H. sosnowskyi*)

| No. | Classes of compounds | Concentration mG g^-1^ DM extract of hogweed parts | | | | |
| --- | --- | --- | --- | --- | --- | --- |
|  |  | Leaves | Stems | Seeds | Flowers | Roots |
| 1 | Alcohols | 1.487 ± 0.015^a^ | 0.1654 ± 0.0050^c^ | n.d. | 1.242 ± 0.028^b^ | 0.2192 ± 0.0010^c^ |
| 2 | Alkanes | 8.934 ± 0.011^b^ | 4.244 ± 0.016^c^ | n.d. | 20.40 ± 0.25^a^ | 8.641 ± 0.157^b^ |
| 3 | Carboxylic acids | 103.2± 1.6^d^ | 76.68 ± 3.92^e^ | 184.0 ± 0.1a | 128.1± 0.8^c^ | 142.9 ± 0.2^b^ |
| 4 | Aromatic carboxylic acids | 1.631 ± 0.170^ab^ | n.d. | 1.900 ± 0.034^ab^ | 0.7855 ± 0.0270^b^ | 4.806 ± 2.000^a^ |
| 5 | Coumarin | 64.48 ± 0.46^d^ | 51.45 ± 0.53^e^ | 198.6 ± 0.6^b^ | 157.3 ± 0.4^c^ | 479.7 ± 0.1^a^ |
| 6 | Terpene | 19.98 ± 0.20^a^ | 1.154 ± 0.032^b^ | n.d. | 1.028 ± 0.066^b^ | 0.919 ± 0.035^b^ |
| 7 | Triterpene | 254.4 ± 0.1^b^ | 130.1 ± 4.0^c^ | 59.50 ± 0.10^d^ | 347.2 ± 4.0^a^ | 65.24 ± 0.85^d^ |

n.d. – Not detected. Of the individual lipid species belonging to different classes, 98 lipids were confirmed by mass spectrometry. The lipids were identified by comparing experimental m/z values with the exact masses of lipids from the literature and by comparing their retention times. In all cases, concentrations were calculated using a series of reference compounds, taking into account differences relative to the class of the identified component.

Table S3. Lipid content (mg 100g^-1^ DW) in different parts of the hogweed (*H. sosnowskyi*)

| No. | Classes of compounds | Concentration mG 100g^-1^ DW parts of hogweed | | | | |
| --- | --- | --- | --- | --- | --- | --- |
|  |  | Leaves | Stems | Seeds | Flowers | Roots |
| 1 | Alcohols | 4.332 ± 0.042^b^ | 0.2373 ± 0.0065^d^ | n.d. | 7.056 ± 0.161^a^ | 0.8508 ± 0.0013^c^ |
| 2 | Alkanes | 26.02 ± 0.03^c^ | 6.087 ± 0.023^d^ | n.d. | 115.9 ± 1.4^a^ | 33.54 ± 0.61^b^ |
| 3 | Carboxylic acids | 300.6 ± 4.6^d^ | 210.0 ± 5.6^e^ | 2104 ± 1^a^ | 727.0 ± 4.5^c^ | 554.8 ± 0.8^b^ |
| 4 | Aromatic carboxylic acids | 4.750 ± 0.494^bc^ | n.d. | 21.83 ± 0.39^a^ | 4.461 ± 0.152^c^ | 18.65 ±7.76^ab^ |
| 5 | Coumarin | 187.8 ± 1.4^d^ | 73.79 ± 0.76^e^ | 2282 ± 87^a^ | 893.5 ± 2.5^c^ | 1862 ± 67^b^ |
| 6 | Terpene | 58.21 ± 0.58^a^ | 1.656 ± 0.046^d^ | n.d. | 5.836 ± 0.377^b^ | 3.567 ± 0.137^c^ |
| 7 | Triterpene | 740.9 ± 11.2^b^ | 186.6 ± 5.7^e^ | 683.6 ± 1.1^c^ | 1972 ± 23^a^ | 253.2 ± 3.3^d^ |

 n.d. – Not detected. Of the individual lipid species belonging to different classes, 98 lipids were confirmed by mass spectrometry. The lipids were identified by comparing experimental m/z values with the exact masses of lipids from the literature and by comparing their retention times. In all cases, concentrations were calculated using a series of reference compounds, taking into account differences relative to the class of the identified component.

Table S4. Names and abbreviation of fatty acids determined in *H.sosnowsky* extracts.

| **No.** | **Fatty acids** (**IUPAC name)** | **Abbreviation** |
| --- | --- | --- |
| 1 | (CLA) (10E,12Z)-octadeca-10,12-dienoate | (CLA) C18:2n-6 (Δ^10t12c^) |
| 2 | Nonanedioic acid | AzA |
| 3 | Dodecanoic acid | C12:0 |
| 4 | Tetradecanoic acid | C14:0 |
| 5 | Pentadecanoic acid | C15:0 |
| 6 | Hexadecanoic acid | C16:0 |
| 7 | (5Z)-Hexadec-5-enoic acid | C16:1n-11 (Δ^5c^) |
| 8 | (12Z)-Hexadec-12-enoic acid | C16:1n-3 (Δ^12c^) |
| 9 | (11Z)-Hexadec-11-enoic acid | C16:1n-5 (Δ^11c^) |
| 10 | (7Z,10Z)-hexadeca-7,10-dienoic acid | C16:2n-6  (Δ^7c10c^) |
| 11 | (9Z)-Hexadec-9-enoic acid | C16:1n-7 (Δ^9c^) |
| 12 | (7Z)-Hexadec-7-enoic acid | C16:1n-9 (Δ^7c^) |
| 13 | (7Z,10Z,13Z)-hexadeca-7,10,13-trienoic acid | C16:3n-3 (Δ^7c10c13c^) |
| 14 | Heptadecanoic acid | C17:0 |
| 15 | (10Z)-Heptadec-10-enoic acid | C17:1n-7 (Δ^10c^) |
| 16 | Octadecanoic acid | C18:0 |
| 17 | (15E)-Octadec-15-enoic acid | C18:1n-2 (Δ^15t^) |
| 18 | (14E)-Octadec-14-enoic acid | C18:1n-3 (Δ^14t^) |
| 19 | (12Z)-Octadec-12-enoic acid | C18:1n-6 (Δ^12c^) |
| 20 | (11Z)-Octadec-11-enoic acid | C18:1n-7 (Δ^11t^) |
| 21 | (9E)-Octadec-9-enoic acid | C18:1n-9 (Δ^9t^) |
| 22 | (10E,12E)-9-oxooctadeca-10,12-dienoic acid | C18:2n-6 (Δ^10t12t^)(oxo-9) |
| 23 | (9Z,12Z)-Octadeca-9,12-dienoic acid | C18:2n-6 (Δ^9c12c^) |
| 24 | (9Z,12E)-Octadeca-9,12-dienoic acid | C18:2n-6 (Δ^9c12t^) |
| 25 | (9Z,12Z,15Z)-Octadeca-9,12,15-trienoic acid | C18:3n-3 (Δ^9c12c15c^) |
| 26 | (11-14E)-Icos-[11-14]-enoic acid | C20:1n-[6-9] (Δ^11-14t^) |
| 27 | (9Z)-Icos-9-enoic acid | C20:1n-11  (Δ^9c^) |
| 28 | (13Z)-Icos-13-enoic acid | C20:1n-7 (Δ^13c^) |
| 29 | (11Z)-Icos-11-enoic acid | C20:1n-9 (Δ^11c^) |
| 30 | Docosanoic acid | C22:0 |
| 31 | Tricosanoic acid | C23:0 |
| 32 | Tetracosanoic acid | C24:0 |
| 33 | Pentacosanoic acid | C25:0 |
| 34 | Hexacosanoic acid | C26:0 |
| 35 | Octanoic acid | C8:0 |
| 36 | 9-Oxononanoic acid | C9:0 9-oxo |
| 37 | 15-Methylhexadecanoic acid | iC17:0 |
| 38 | Butanedioic acid | Suc |
| 39 | Decanoic acid | C10:0 |

Table S5. Lipid yield results from *Heracleum sosnowskyi* extract and parameters for creating a box diagram.

| Number of repetitions | Extract yield by different extraction methods, mg g^-1^ DW | | | |
| --- | --- | --- | --- | --- |
|  | Convencional | Ultrasound | Accelerated solv. | Soxhlet |
| 1 | 136.69 | 83.11 | 70.15 | 179.47 |
| 2 | 134.25 | 79.41 | 77.23 | 180.26 |
| 3 | 142.54 | 89.12 | 73.41 | 188.67 |
| 4 | 134.47 | 82.66 | 75.36 | 175.72 |
| 5 | 130.34 | 81.01 | 78.55 | 183.18 |
| Parameters |  |  |  |  |
| Min | 130.34 | 79.41 | 70.15 | 175.72 |
| Quartile1 | 132.30 | 80.21 | 71.78 | 177.60 |
| Median | 134.47 | 82.66 | 75.36 | 180.26 |
| Quartile3 | 139.62 | 86.12 | 77.89 | 185.93 |
| Max | 142.54 | 89.12 | 78.55 | 188.67 |
| Mean | 135.66 | 83.06 | 74.94 | 181.46 |
| IQR (interquartile range) | 7.32 | 5.91 | 6.11 | 8.33 |
| Lower outlier limit | 121.32 | 71.35 | 62.62 | 165.10 |
| Upper outlier limit | 150.60 | 94.97 | 87.06 | 198.42 |

Table S6. Free fatty acid composition of the extract of the studied parts of *H. sosnowskyi* using GC-MS analysis

| **Fatty acid** | **Classification of Fatty Acids and Corresponding Values** | **RI** | **Content of free fatty acids, µg 100 dg^-1^ DM** | | | | | |
| --- | --- | --- | --- | --- | --- | --- | --- | --- |
|  |  |  | **Roots** | **Stems** | **Leaves** | **Flowers** | **Immature seeds** | **Mature seeds** |
| (CLA) C18:2n-6 (Δ^10t,12c^) | 4 | 2604 | <0.1 | <0.1 | <0.1 | <0.1 | <0.1 | 22.5 ± 0.3 ^a^ |
| C17:1n-7 (Δ^10c^) | 2 | 2288 | 3.0 ± 0.1 ^b^ | <0.1 | <0.1 | 134.8 ± 3.0 ^a^ | 0.1 ± 0.0 ^b^ | 2.2 ± 0.2 ^b^ |
| C20:1n-9 (Δ^11c^) | 2 | 2673 | 6.4 ± 0.1 ^d^ | 22.4 ± 0.3 ^c^ | 52.4 ± 4.8 ^b^ | 247.5 ± 6.3 ^a^ | 1.2 ± 0.0 ^d^ | 3.5 ± 0.1 ^d^ |
| C16:1n-5 (Δ^11c^) | 2 | 2284 | 3.8 ± 0.1 ^c^ | 4.2 ± 0.0 ^b^ | <0.1 | 10.1 ± 0.4 ^a^ | <0.1 | <0.1 |
| C16:1n-3 (Δ^12c^) | 2 | 2288 | 3.1 ± 0.0 ^c^ | 53.9 ± 3.3 ^b^ | 409.9 ± 16.0 ^a^ | 37.4 ± 0.5 ^b^ | <0.1 | 1.0 ± 0.0 ^c^ |
| C18:1n-6 (Δ^12c^) | 2 | 2479 | 68.3 ± 4.9 ^ab^ | 51.4 ± 1.1 ^b^ | 75.8 ± 3.5 ^a^ | 54.8 ± 5.2 ^ab^ | <0.1 | 22.8 ± 19.7 ^c^ |
| C20:1n-7 (Δ^13c^) | 2 | 2683 | <0.1 | 15.2 ± 0.6 ^b^ | 6.7 ± 5.8 ^bc^ | 192.6 ± 5.5 ^a^ | 1.7 ± 0.0 ^c^ | 3.2 ± 0.1 ^c^ |
| C16:1n-11 (Δ^5c^) | 2 | 2256 | 5.2 ± 0.1 ^d^ | <0.1 | 8.7 ± 0.1 ^b^ | 18.0 ± 0.3 ^a^ | 0.9 ± 0.0 ^e^ | 5.8 ± 0.0 ^c^ |
| C16:3n-3 (Δ^7c,10c,13c^) | 4 | 2370 | 8.4 ± 0.0 ^d^ | 433.6 ± 14.1 ^b^ | 3017.0 ± 28.1 ^a^ | 217.4 ± 2.1 ^c^ | <0.1 | <0.1 |
| C16:2n-6 (Δ^7c,10c^) | 3 | 2303 | 2.1 ± 0.0 ^c^ | 30.9 ± 0.6 ^a^ | 25.5 ± 0.0 ^b^ | 31.0 ± 0.2 ^a^ | <0.1 | <0.1 |
| C16:1n-9 (Δ^7c^) | 2 | 2261 | 7.6 ± 0.1 ^cd^ | 178.0 ± 7.3 ^b^ | 13.2 ± 0.1 ^c^ | 244.7 ± 1.4 ^a^ | 1.0 ± 0.0 ^d^ | 7.1 ± 0.1 ^cd^ |
| C20:1n-11 (Δ^9c^) | 2 | 2668 | <0.1 | <0.1 | <0.1 | <0.1 | 0.9 ± 0.0^b^ | 7.3 ± 0.3 ^a^ |
| C16:1n-7 (Δ^9c^) | 12.5 | 2270 | 4.8 ± 0.1 ^c^ | 20.2 ± 0.1 ^b^ | 33.5 ± 0.1 ^a^ | 20.1 ± 0.1 ^b^ | 0.9 ± 0.0 ^e^ | 1.5 ± 0.1^d^ |
| AzA | 1 | 2154 | 14.6 ± 0.1 ^d^ | 80.2 ± 0.6 ^a^ | 22.7 ± 0.1 ^c^ | 42.5 ± 0.4 ^b^ | 0.4 ± 0.3 ^e^ | <0.1 |
| C22:0 | 1 | 2863 | 25.7 ± 0.2 ^d^ | 186.1 ± 14.6 ^b^ | 116.1 ± 1.4 ^c^ | 229.1 ± 1.0 ^a^ | 3.8 ± 0.0 ^e^ | 10.0 ± 0.4 ^de^ |
| C8:0 | 2 | 1398 | 6.4 ± 0.3 ^c^ | 12.2 ± 0.2^b^ | <0.1 | 25.4 ± 0.2^a^ | 2.4 ± 0.0 ^d^ | 2.1 ± 0.1^d^ |
| C18:1n-7 (Δ^11t^) | 2 | 2474 | 22.1 ± 0.4^cd^ | 336.7 ± 0.6 ^b^ | 45.0 ± 4.2 ^c^ | 359.6 ± 4.8 ^b^ | 2.9 ± 0.0 ^d^ | 2177.1 ± 26.4 ^a^ |
| C20:1n-[6-9] (Δ^14-11t^) | 2 | 2652 | 4.7 ± 0.0 ^d^ | 223.5 ± 1.4^b^ | 15.4 ± 0.0 ^c^ | 237.3 ± 8.8 ^a^ | 2.4 ± 0.0 ^d^ | 7.4 ± 0.0 ^cd^ |
| C18:1n-9 (Δ^9t^) | 1 | 2468 | 85.8 ± 0.5 ^e^ | 119.9 ± 1.9 ^d^ | 368.0 ± 8.9^b^ | 1946.6 ± 3.6 ^a^ | 259.1 ± 0.2 ^c^ | <0.1 |
| C26:0 | 1 | 3260 | 20.0 ± 0.4^d^ | 163.2 ± 3.4 ^b^ | 197.7 ± 0.0 ^a^ | 88.8 ± 2.5^c^ | 0.8 ± 0.0 ^e^ | <0.1 |
| iC17:0 | 4 | 2306 | <0.1 | 4.1 ± 0.0 ^a^ | <0.1 | <0.1 | <0.1 | <0.1 |
| C18:2n-6 (Δ^10t,12t^)(oxo-9) | 1 | 3185 | <0.1 | 33.3 ± 1.5 ^a^ | <0.1 | <0.1 | <0.1 | <0.1 |
| C12:0 | 3 | 1817 | 3.7 ± 0.0 ^d^ | 13.1 ± 0.1 ^c^ | 19.6 ± 0.1 ^b^ | 50.4 ± 0.1 ^a^ | 0.1 ± 0.0 ^f^ | 0.5 ± 0.0 ^e^ |
| C18:2n-6 (Δ^9c,12c^) | 3 | 2521 | 1674.6 ± 17.5 ^c^ | <0.1 | 4065.4 ± 167.9 ^b^ | 7998.2 ± 102.0 ^a^ | <0.1 | 1037.7 ± 11.1^d^ |
| C18:2n-6 (Δ^9c,12t^) | 4 | 2515 | 50.1 ± 3.2 ^b^ | 4736.9 ± 103.9 ^a^ | 47.1 ± 4.0 ^b^ | 68.1 ± 1.2 ^b^ | 120.0 ± 0.9 ^b^ | 5.0 ± 0.2 ^b^ |
| C18:3n-3 (Δ^9c,12c,15c^) | 1 | 2581 | 209.3 ± 7.5 ^d^ | 563.1 ± 8.4 ^c^ | 8039.2 ± 93.3 ^a^ | 4701.5 ± 19.6 ^b^ | 2.2 ± 0.2 ^e^ | 17.4 ± 0.0 ^e^ |
| C17:0 | 1 | 2339 | 13.7 ± 0.0 ^d^ | 119.3 ± 1.1 ^b^ | 80.5 ± 0.8 ^c^ | 127.5 ± 1.3 ^a^ | 0.7 ± 0.0 ^e^ | 2.6 ± 0.0 ^e^ |
| C14:0 | 2.5 | 2025 | 14.6 ± 0.1 ^c^ | 155.8 ± 0.7 ^b^ | 157.7 ± 1.4 ^b^ | 597.0 ± 3.9 ^a^ | 1.4 ± 0.0 ^d^ | 3.5 ± 0.0 ^d^ |
| C9:0 9-oxo | 1 | 2077 | 12.7 ± 0.0 ^bc^ | 183.2 ± 22.7 ^a^ | 16.0 ± 0.1 ^bc^ | 25.5 ± 0.1 ^b^ | <0.1 | <0.1 |
| C16:0 | 1 | 2236 | 543.3 ± 3.1 ^d^ | 6977.7 ± 12.9 ^a^ | 2933.7 ± 12.3 ^c^ | 4764.7 ± 23.8 ^b^ | 28.1 ± 0.1 ^f^ | 205.0 ± 2.8 ^e^ |
| C25:0 | 1 | 3174 | 100.8 ± 0.4 ^a^ | 44.4 ± 2.4 ^b^ | 49.2 ± 4.1 ^b^ | 29.9 ± 2.9 ^c^ | <0.1 | 1.2 ± 0.0 ^d^ |
| C15:0 | 1 | 2131 | 25.0 ± 1.3 ^d^ | 148.5 ± 0.4 ^a^ | 38.0 ± 1.3 ^c^ | 82.4 ± 0.2 ^b^ | 0.5 ± 0.0 ^e^ | 2.3 ± 0.0 ^e^ |
| C18:0 | 2 | 2450 | 47.7 ± 0.5 ^d^ | 357.8 ± 0.7 ^c^ | 404.1 ± 11.9 ^b^ | 653.2 ± 19.5 ^a^ | 9.4 ± 0.1 ^e^ | 55.2 ± 0.4 ^d^ |
| Suc + C10:0 | 1 | 1609 | 2.5 ± 0.0 ^d^ | 25.6 ± 0.1 ^a^ | 7.6 ± 0.1 ^c^ | 9.9 ± 0.2 ^b^ | <0.1 | <0.1 |
| C24:0 | 1 | 3070 | 47.1 ± 0.1 ^c^ | 468.2 ± 32.0 ^a^ | 290.8 ± 41.7 ^b^ | 473.5 ± 41.7 ^a^ | <0.1 | <0.1 |
| C18:1n-3 (Δ^14t^) | 2 | 2490 | 10.9 ± 1.3 ^c^ | 20.6 ± 0.5 ^a^ | 10.3 ± 1.4 ^c^ | 17.3 ± 1.4 ^b^ | 5.3 ± 0.7 ^d^ | 6.8 ± 0.1 ^d^ |
| C18:1n-2 (Δ^15t^) | 2 | 2501 | 67.8 ± 0.7 ^b^ | 39.8 ± 4.7 ^c^ | 42.5 ± 1.8 ^c^ | 91.8 ± 2.5 ^a^ | <0.1 | <0.1 |
| C23:0sS | 1 | 2965 | 10.7 ± 0.9 ^d^ | 134.2 ± 8.8 ^a^ | 63.7 ± 5.2 ^c^ | 118.0 ± 2.5 ^b^ | <0.1 | <0.1 |
|  | | | 290.9 ± 6.7^e^ | 893.2 ± 11.9^d^ | 1091.5 ± 11.8^c^ | 3406.3 ± 17.8^a^ | 274.0 ± 0.9^e^ | 2238.2 ± 16.2^b^ |
|  | | | 1944.4 ± 20.4^d^ | 5797.7 ± 120.2^c^ | 15194.3 ± 240.5^a^ | 13016.2 ± 116.4^b^ | 122.2 ± 0.8^f^ | 1082.5 ± 11.1^e^ |
|  | | | 893.4 ± 3.3^d^ | 9297.1 ± 68.5^a^ | 4412.9 ± 66.0^c^ | 7555.0 ± 55.1^b^ | 49.9 ± 0.4^f^ | 289.8 ± 3.0^e^ |
| UFA/SFA | | | 0.7 ± 1.5 | 3.6 ± 3.8 | 2.7 ± 7.2 | 7.6 ± 2.4 | 11.5 ± 10.7 | 2.2 ± 1.4 |
| Unsaturation index | | | 84.3 | 203.6 | 138.1 | 116.0 | 122.4 | 143.4 |

Data are expressed as the mean ± SD; RI – Retention index for Omegawax250 capillary column (SD ± 3); superscripts (a, b, c, d, e, f) - represent the significance of differences between values using the Tukey HSD test (p < 0.05); MUFA - monounsaturated fatty acid; PUFA - polyunsaturated fatty acid; SFA – saturated fatty acids; AzA – azelaic acid; Suc – Succinic acid.  Classification of Fatty Acids and Corresponding Values - Saturated has a value of 1, Monounsaturated – 2, Diunsaturated – 3, Triunsaturated – 4, AzA - 12.5, CLA – 4.


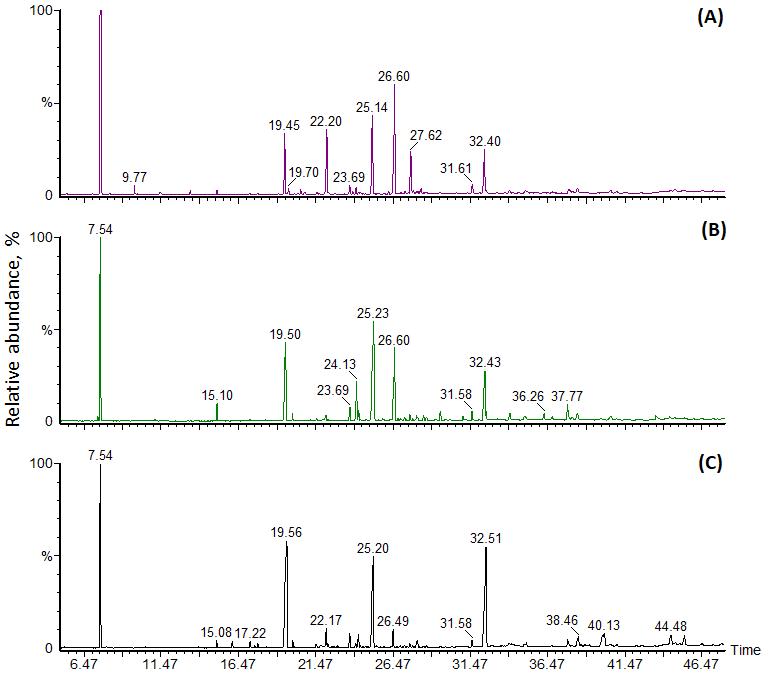


Fig. S1. GC-MS chromatograms of fatty acid methyl esters (FAME) of *H. sosnowskyi* samples obtained from extracts of plant parts such as leaves (A), flowers (B), and stems (C).


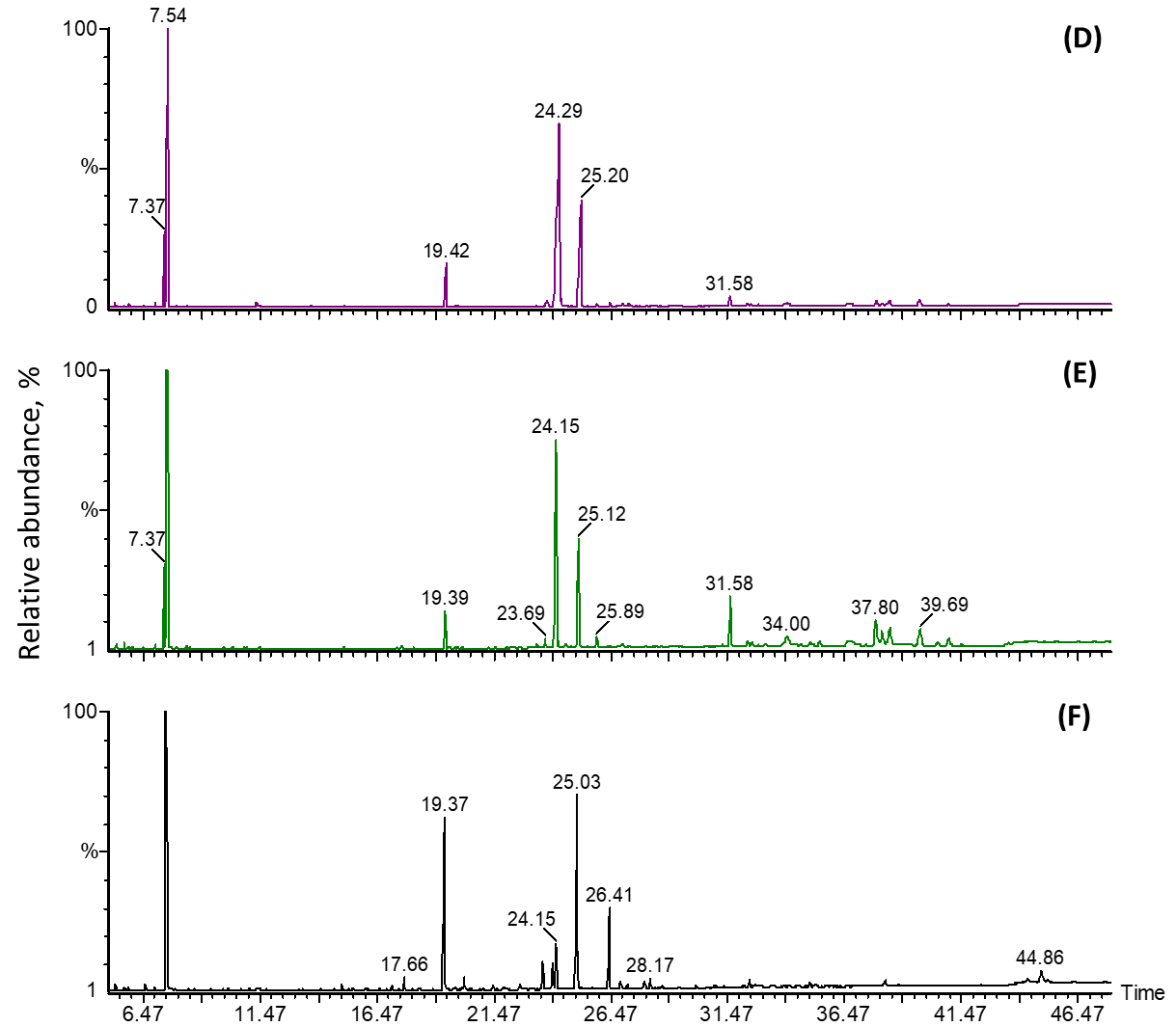


Fig. S2. GC-MS chromatograms of fatty acid methyl esters (FAME) of *H. sosnowskyi* samples obtained from extracts of plant parts such as mature seeds (D), immature seeds (E), and roots (F).
